# Developing CHARMM Force Field Parameters for the Radical States of Tryptophan and Tyrosine

**DOI:** 10.1021/acs.jctc.6c01280

**Published:** 2026-09-01

**Authors:** Andrei Y. Kostritski, Jonathan Hungerland, Christian Schuster, Ilia A. Solov’yov

**Affiliations:** † Institute of Physics, School of Mathematics and Science, 11233 Carl von Ossietzky Universität Oldenburg, Carl-von-Ossietzky Str. 9-11, Oldenburg 26111, Germany; ‡ Research Center for Neurosensory Sciences, Carl von Ossietzky Universität Oldenburg, Carl-von-Ossietzky Str. 9-11, Oldenburg 26111, Germany; § Institute of Physics, Center for Nanoscale Dynamics (CENAD), Carl von Ossietzky Universität Oldenburg, Oldenburg 26129, Germany

## Abstract

Amino acid radicals play fundamental roles in many physiological processes ranging from DNA repair and synthesis to respiration and magnetoreception. In particular, tryptophan and tyrosine in their radical states often serve as key intermediates in electron transfer processes. Importantly, the radical states can be long-lived enough to trigger conformational changes in proteins that can be important for biomolecular signaling. Molecular dynamics (MD) simulations provide a perfect means to study the effects of tyrosine and tryptophan oxidation on the conformational dynamics of proteins and to reveal molecular mechanisms of their biological roles. However, a reliable application of MD simulations requires a comprehensive set of molecular mechanics parameters to describe amino acid residues in the radical states. Here, we present a complete set of molecular mechanics parameters for the radical states of tyrosine and tryptophan residues, developed within the framework of the CHARMM force field. Optimized to target a large body of quantum mechanical data, the parameters provide a robust description of the intramolecular energetics, interactions with water, and electrostatic properties of tyrosine and tryptophan in their radical states. The parameters were tested and validated through simulations of model protein systems including the α_3_W protein, a β-hairpin maquette, and an avian cryptochrome. The simulation results demonstrate the effects of tyrosine and tryptophan oxidation on side-chain dynamics, conformational changes, and general biophysical properties of proteins. We expect that the presented set of force field parameters would be useful for the computational studies of various biological processes that involve oxidation of the tyrosine and tryptophan residues.

## Graphical abstract

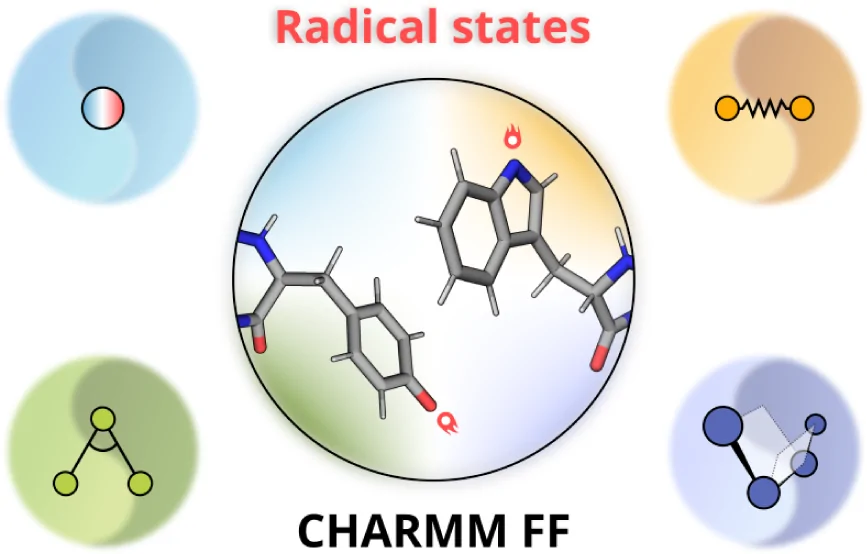

## Introduction

Amino acid radicals play important roles in various physiological processes^1,2^ including biosynthesis^3^ and repair^4,5^ of DNA, photosynthesis^6,7^ and magnetoreception.^8–10^ Formed as a result of one-electron oxidation, radicals of cysteine, glycine, tyrosine (TYR), and tryptophan (TRP) can serve as key intermediates in electron transfer (ET) and proton-coupled ET (PCET) processes.^1^ In particular, aromatic TYR and TRP residues can form extended chains thereby enabling multistep ET that is essential for long-distance ET/PCET.^1,11^ Such chains are particularly important for DNA synthesis and repair facilitated by ribonucleotide reductases^12^ and photolyases,^13^ and for the formation of the long-lived radical pairs that are critical for the efficient magnetoreception mediated by cryptochromes.^8,14^ The abundance and efficiency of TYR and TRP radicals in mediating ET have also motivated the development of model protein systems to study their redox properties in a more controlled manner^15–17^ revealing key thermodynamic and kinetic properties of ET/PCET in proteins.^1^ In particular, model systems such as the α_3_Y and α_3_W proteins^16^ have been used to demonstrate how the standard redox potential of the TYR and TRP residues is shifted by the protein matrix.^17–19^

Whereas the protein environment affects the electrochemical properties of amino acid residues,^18,20^ a change in the redox state of a residue can, in turn, affect local biophysical properties^21^ and the overall dynamics of the protein.^22,23^ Importantly, TYR and TRP radicals can be rather long-lived with lifetimes ranging from milliseconds^18,19^ to hours,^24,25^ inducing protein conformational changes that are important for biomolecular signaling.^26^ Molecular dynamics (MD) simulations provide a perfect means to study molecular mechanisms of the signaling processes mediated by proteins.^27^ The proper use of MD requires, however, a thorough optimization of the underlying molecular-mechanics (MM) parameters. Although tools such as the Force Field Toolkit enable the basic parametrization of a wide range of molecules,^28,29^ parametrizing nonstandard amino acids and radical species often requires a more elaborate approach.^30,31^ Therefore, the development of MM parameters for the TYR residue in the neutral radical state and the TRP residue in the cationic radical state has been so far limited to the optimization of atomic partial charges.^10,17,22,23^ However, studying the effects of TYR and TRP oxidation on long-timescale protein dynamics also requires optimization of the bonded parameters including the dihedral parameters that play a fundamental role in protein conformational changes. Finally, to the best of our knowledge, no MM parameters are presently available to describe TRP in the neutral radical state or TYR in the cationic radical state.

Here we introduce the full set of MM parameters for the TYR and TRP residues in their neutral and cationic radical states within the framework of the CHARMM force field.^32^ The parameters for bonded interactions were optimized to reproduce extensive potential energy scans at the quantum mechanical (QM) level of theory. Particular attention was paid to the optimization of side-chain dihedral interactions that are crucial for the correct simulations of long-timescale protein dynamics. Atomic partial charges were optimized to reproduce the interactions of model compounds with water molecules, their dipole moments, as well as the electrostatic potential obtained at the QM level of theory. The resulting set of force field parameters was tested in simulations of model proteins such as peptide A^15^ and α_3_W,^16^ and avian cryptochrome^33^ to demonstrate the effects of amino acid radicals on the biophysical properties and dynamics of the proteins.

## Methods

### Optimization of Partial Charges

Model compounds 3-methylindole (3MI) and 4-methylphenol (4MP) were used to derive atomic partial charges of the side chains of TRP and TYR, respectively. The parametrization procedure largely followed the original CHARMM protocol,^32^ that is focused on reproducing interaction energies and optimal distances between water molecules and a model compound as well as its dipole moment calculated at the QM level of theory. To foster transferability of the resulting force field parameters, the electrostatic potential around the model compounds was calculated at the QM level of theory and was also included in the optimization function, as proposed in the GAAMP approach^34^ and recently used for the parametrization of nonstandard amino acid residues.^31^ Thus, the optimization procedure was based on minimizing the following loss function:

$$\begin{array}{ll} L & =w_{E}\overset{Nconf}{\underset{i}{\sum }}{\left(E_{i}^{MM}-E_{i}^{QM}\right)}^{2}+w_{R}\overset{Nconf}{\underset{i}{\sum }}{\left(R_{i}^{MM}-R_{i}^{QM}\right)}^{2}\\ & +w_{\mu }\overset{3}{\underset{i}{\sum }}{\left(\mu_{i}^{MM}-\mu_{i}^{QM}\right)}^{2}+w_{\phi }\overset{N_{p}}{\underset{i}{\sum }}{\left(\phi_{i}^{MM}-\phi_{i}^{QM}\right)}^{2}+w_{Q}{\left(\overset{Natoms}{\underset{i}{\sum }}q_{i}-Q_{tot}\right)}^{2} \end{array}$$ 1

where the first two terms account for the interaction energy (*E*_*i*_) and the equilibrium distance (*R*_*i*_) between a model compound and a single water molecule in the *i*th water–compound configuration, the third term accounts for the dipole moment (μ), the fourth term accounts for the electrostatic potential (ϕ) around the model compound, and the last term ensures the correct total charge of the compound. The summation in the first two terms goes over multiple water–compound configurations probing different interaction sites as shown in Figure S1. The three components of the dipole moment were optimized independently, resulting in the concomitant optimization of the angle between the dipole moments calculated at the MM and QM levels of theory. The QM superscript denotes reference values calculated at the QM level of theory, in contrast to the MM values that varied during the charge optimization. Finally, the weights (*w*_*E*_, *w*_*R*_, *w*_μ_, *w*_ϕ_, *w*_*Q*_) were used as hyperparameters to tune the scaling and relative contributions of the different terms to the loss function. Minimization of the loss function was done with the steepest-descent algorithm until a threshold of 0.005 was reached in the loss-function change between two successive optimization cycles. The final charges contained three decimal digits, with the last digit adjusted such that the total charge was an integer. All QM calculations were conducted with ORCA version 5.^35,36^

Before charge optimization, the geometry of the compounds was optimized, employing the B3LYP functional^37^ with the 6-31G* basis set.^38^ To follow the original CHARMM protocol,^32^ energies and optimal distances of the water–compound configurations were calculated at the Hartree–Fock (HF) level of theory^39,40^ employing the 6-31G* basis set. The restricted–open-shell HF approach was utilized to prevent spin contamination. Computed interaction energies (*E* ^*QM*^) of neutral compounds with water were multiplied by 1.16, and a value of 0.2 Å was subtracted from the interaction distances (*R* ^*QM*^) to account for the lack of multibody polarization effects.^32^ Upon geometry optimization of the water-compound configurations, only the distance *R* was free to change, whereas the orientation of the water molecules was kept restrained.

The dipole moment (μ^*QM*^) was calculated with the HF method and the 6-31G* basis set. The resulting value was multiplied by a factor of 1.35 to account for the lack of medium polarizability in the QM calculations conducted in vacuo.^41^ In the case of charged compounds, the dipole moment is ill-defined and, therefore, was excluded from the loss function. The electrostatic potential (ϕ^*QM*^) was calculated at the same level of theory at *N*_*p*_ (∼1300) points around the compound. Following the GAAMP approach,^34^ the points were distributed over five layers with a density of 1 point per Å^2^ in each layer. The layers were located at varying distances from the compound, that were defined by the atomic vdW radii multiplied by factors ranging from 1.4 to 2.2. The distribution of points was generated with the DMS program as implemented in the Chimera package.^42^

### Bond and Angle Parameter Optimization

The potential energy associated with bonds and valence angles is defined as^32^

2 $$V=k{\left(x-x_{0}\right)}^{2}$$

Here *x* denotes the bond length or angle, *x*_0_ is its equilibrium value, and *k* is the force constant. The *x*_0_ and *k* parameters for bonded interactions were optimized to reproduce potential energy scans (PES) calculated at the QM level of theory. The PES were obtained with the B3LYP functional and the 6-31G* basis set, largely following the workflow that was utilized for recently parametrized compounds in the CHARMM force field.^30,31,43^ In particular, relaxed PES for each degree of freedom were conducted over seven points around the equilibrium value, such that the potential energy at the maximum deformation was around 8.36 kJ/mol. Optimization of the bond and angle parameters was conducted simultaneously, to account for their interdependence in conjugated systems such as the side chains of the TYR and TRP residues. As MP2 is a canonical method for deriving bonded parameters in the original CHARMM force field,^32,41^ some PES were also computed at the MP2 level of theory, and compared to the B3LYP results. In this case, the restricted–open-shell HF approach was employed to prevent spin contamination.

### Dihedral Parameter Optimization

The potential energy associated with the dihedral interactions is defined as^32^

3 $$V_{d}=\underset{n}{\sum }k_{n}\left(1+\cos\left(n\phi -\phi_{0}\right)\right)$$

Here *k*_*n*_ is the force constant for the term with multiplicity *n*, ϕ denotes the dihedral angle, and ϕ_0_ is the offset, usually set to either 0° or 180°. Depending on whether the four atoms constituting the dihedral form a planar group, the dihedrals can be split into stiff and soft categories. The stiff dihedrals are characterized by a steep increase in the potential energy around a single potential energy minimum at either 0° or 180°, and therefore can be described by a single term with multiplicity *n* = 2. The parameters of the stiff dihedrals were optimized for 3MI and 4MP similar to the bond and angle parameters.

In contrast, soft dihedrals usually have multiple local minima in their potential energy landscape and are described by multiple terms in eq 3. In the TYR and TRP residues, soft dihedrals are limited to the χ_1_ and χ_2_ side-chain angles that were optimized using the dipeptide models with TYR and TRP capped with the acetyl and *N*-methylamine groups. It is important to note that a rotational scan of the side chains can lead to changes in the backbone conformation, which has to be restrained to ensure targeted optimization of the parameters for the selected dihedrals. Although parametrization of the amino acid side chains is often done only with the α-helical conformation of the backbone,^31,32^ we noticed that such an approach could lead to parameters that are suboptimal for alternative backbone conformations. Therefore, we used multiple backbone conformations of the dipeptides to cover a larger part of the Ramachandran plot. In addition to the α-helical conformation (*alpha*), we considered the β-strand conformation (*beta*) defined by the backbone dihedral angles ϕ = −140° and ψ = 135°. Furthermore, we used the backbone conformations that correspond to the collagen helix^44^ (*collagen*). In the *collagen* conformation, the exact values of the backbone ϕ and ψ angles were slightly different for the TRP and TYR dipeptides and corresponded to the backbone conformations of residues W318 and Y319 as observed in the crystal structure of the pigeon (*Columba livia*) cryptochrome (ClCry4).^33^ The corresponding backbone dihedral angles were ϕ = −60° and ψ = 152° in the TRP dipeptide, and ϕ = −70° and ψ = 140° in the TYR dipeptide. Finally, we also included the backbone conformation of the left-handed *alpha* helix (*l* - *handed*) with the backbone dihedral angles ϕ = 60° and ψ = 38°. This backbone conformation corresponds to that of residue W369 in ClCry4. For each backbone conformation, a 360° PES was performed for the χ_1_ and χ_2_ dihedral angles, with all other dihedral angles restrained to the optimized geometry. The resulting QM scans were simultaneously targeted by the multiconformation fitting procedure, employing the Monte Carlo simulated annealing algorithm.^45^ To focus on the lower-energy conformations of the dipeptides, PES points with energy values higher than 42 kJ/mol were excluded from the fitting. Furthermore, the relative weights of different backbone conformations were varied to optimize the overall fitting quality. In particular, the contribution of the *beta*-conformation scans was weighted by 0.25 when fitting the PES of the TRP dipeptide models, and the contributions of the *alpha*- and *beta*-conformation scans were weighted by 0.5 when fitting the PES of the TYR dipeptide in the neutral radical state. Equal weights were assigned to the backbone conformations in the cationic radical state of the TYR dipeptide. All dihedral angles that were not subject to the optimization were restrained during the MM and QM PES to the values observed in the optimized geometry of the dipeptide.

### Other Parametrization Details

We used parameters of TYR and TRP in the nonradical state from the GROMACS port (July 2022) of the CHARMM force field^46^ as initial parameters for the radical states, which were subjected to optimization. PES for bonds, valence angles, and stiff dihedral angles at the MM level were conducted with GROMACS^47^ version 2019. To include the resulting bonded parameters in the CHARMM force field, we introduced new atom types, whose vdW parameters were taken directly from their nonradical counterparts in the original CHARMM36m force field.^48^

### Simulation Details

The developed parameters were validated in several benchmark simulations of the model proteins peptide A and α_3_W, and of the avian cryptochrome ClCry4. The structure of peptide A was predicted by AlphaFold 3 using the AlphaFold web server,^49^ the NMR structure^50^ (PDB ID: 1LQ7) was used to simulate α_3_W, and ClCry4 was modeled based on the X-ray crystal structure (PDB ID: 6PU0).^33^ In α_3_W protein models, the N- and C-termini were capped with acetyl and *N*-methylamine, respectively. Note that the PDB-based residue numbering of α_3_W used here differs by 2 from the original α_3_ numbering.^51^ The standard protonation state at neutral pH was assigned to all residues of α_3_W and peptide A. In the ClCry4 model, histidines H3 and H7 were considered doubly protonated.^52^

MD simulations of peptide A and α_3_W were performed using GROMACS^47^ version 2025, with a time step of 2 fs. A pressure of 1 bar was applied isotropically with a time constant of 5 ps, using the c-rescale barostat.^53^ The temperature was maintained at 300 K with the v-rescale thermostat,^54^ using a time constant of 0.5 ps. van der Waals interactions were modeled with the Lennard-Jones potential and a cutoff radius of 1.2 nm, with forces smoothly switched to zero in the range of 1.0–1.2 nm without a dispersion correction. Electrostatic interactions were treated by the smooth particle mesh Ewald (SPME) method^55,56^ with a real-space cutoff distance of 1.2 nm. The standard residues in the proteins were described by the CHARMM36m^48^ force field. All hydrogen-involving bonds were constrained using the LINCS algorithm.^57^ Ions were described using CHARMM parameters with the NBFIX correction,^58^ and the CHARMM TIP3P model was used for water molecules. The peptide A and α_3_W systems were simulated in cubic boxes with edge lengths of ∼5.5 and 7 nm, respectively. Na^+^ and Cl^–^ were added to the solution with a bulk concentration of 100 mM and 150 mM in the peptide A and α_3_W systems, respectively. All simulations were conducted in three replicas using different starting velocities in the peptide A system, and using different NMR models as starting structures in the case of α_3_W. The α_3_W and peptide A systems were equilibrated in two steps. The system was first simulated for 5 ns with position restraints applied to all heavy atoms of the protein, followed by 10 ns simulations with the restraints applied only to the backbone atoms. After equilibration, the peptide A and α_3_W systems were simulated without restraints for 1000 ns and 500 ns, respectively.

Simulations of ClCry4 were performed using NAMD^59,60^ as previously described.^52,61^ Briefly, the simulations were run at a temperature of 310 K and a pressure of 1 bar maintained with the Langevin thermostat and the Langevin piston method,^62^ respectively. The protein was simulated in a water solution with 50 mM NaCl. The FAD cofactor was simulated in the anionic semiquinone state with the previously developed parameters.^10^ The pre-equilibrated ClCry4 systems were taken from a previous study^52^ and simulated for 400 ns.

GROMACS tools,^47^ including the DSSP implementation,^63^ were used to analyze structural properties of the proteins. The *g*_*elpot* tool^64^ with its extension for single-residue calculations^65^ was used to quantify the electrostatics of α_3_W. The electrostatic potential of the GLU and LYS residues was calculated at the geometric center of the carboxyl and amino groups, respectively. Analysis of the librational motions in TRP was done using the MDAnalysis library.^66^ All visualizations were done using PyMOL version 1.8.

## Results and Discussion

The section is organized as follows: we first present the optimization of partial charges and the parametrization of bond, angle, and stiff-dihedral energy terms, followed by the optimization of the soft-dihedral parameters for the TYR and TRP residues in their radical states; we then assess and validate the resulting parameters through MD simulations of model protein systems.

### Charge Optimization

We optimized the partial charges of the side-chain atoms of the TRP and TYR residues in their radical states using 3-methylindole (3MI) and 4-methylphenol (4MP) as model compounds. Figure 1 shows the chemical structures of the model compounds, with their atoms labeled according to the CHARMM naming scheme. The geometry of the model compounds was first optimized at the QM level of theory, followed by probing their interaction with water. Multiple water–compound configurations were used to extract interaction energies (*E*) and distances (*R*) between the compound and a single water molecule (Figure S1). The energies and distances were targeted by the optimization procedure (see Methods), where the root-mean-square deviation (RMSD) between MM and QM energies and distances over all the water–compound configurations was used to estimate the quality of the charge optimization (Table 1). The MM energies are in a good agreement with the reference QM data, with the RMSD approaching the thermal energy *k*_*B*_ *T* (∼2.5 kJ/mol at 300 K) for the charged compounds, and going below *k*_*B*_ *T* for the neutral compounds. The QM distances are also well reproduced with the new charges for the neutral compounds, with the RMSD being below 0.1 Å. In the case of the cationic compounds, the distance RMSD is generally higher and reaches the level of around 0.2 Å, which is common for parametrization of charged compounds in the CHARMM framework.^41^ Overall, the new partial charges ensure a proper description of the interaction between the side chains of the TYR and TRP residues and water molecules in the classical MD simulations.

**Figure 1:**
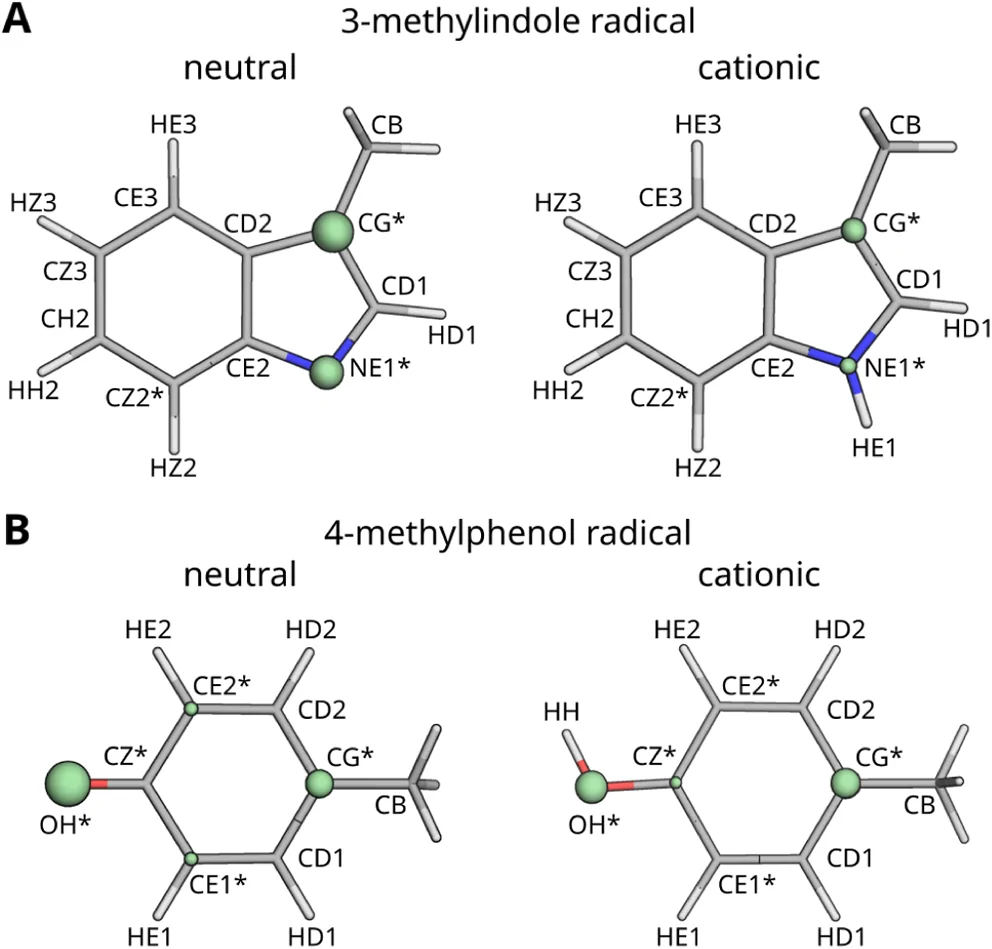
Model compounds used for the optimization of partial charges and parameters for bonds, angles, and stiff dihedrals. **A** 3-Methylindole radical was used to parametrize TRP in the neutral and cationic radical states. **B** 4-Methylphenol was used to parametrize TYR in the neutral and cationic radical states. Atoms are labeled with their atom names. The asterisks mark atoms with newly introduced atom types. The spin density is shown in green and contoured at 0.27 Å^–3^.

**Table 1:** Optimization of Partial Charges

	3-methylindole radical		4-methylphenol radical	
	neutral	cationic	neutral	cationic
E (kJ/mol)^a^	0.77/2.15	2.64/4.94	1.53/3.73	2.61/4.04
R (Å)^a^	0.03/0.08	0.20/0.33	0.10/0.27	0.21/0.30
dipole moment^b^	4.12/3.96/0.0	4.70/4.50/1.2	6.69/6.20/0.0	2.29/3.22/0.6
ϕ (kJ/mol/e)^a^	9.79/42.50	6.83/28.28	11.52/35.34	9.52/46.90

a Data are shown as RMSD/Max |err|, where RMSD is the root-mean-square deviation and Max |err| is the maximum absolute error in reproducing water-compound energies (E), water-compound optimal distances (R), and electrostatic potential of the compound (ϕ) calculated at the QM level of theory by the optimized partial charges.

b Data are shown as |μ^QM^|/|μ^MM^|/θ, where |μ^QM^| is the magnitude of the dipole moment calculated at the QM level of theory and scaled by a factor of 1.35, |μ^MM^| is the magnitude of the dipole moment created by the optimized partial charges, and θ is the angle between the QM and MM dipole moments. |μ| is expressed in Debye units. Note that the dipole moment was targeted only in the charge optimization of the neutral compounds.

The optimized charges reproduce well the magnitude and the direction of the reference QM dipole moment (μ) of the neutral compounds, and of 3MI in the cationic radical state, even though the dipole moment of the latter was not targeted during optimization. However, the lack of dipole moment optimization resulted in overestimation of the dipole moment in the case of 4MP in the cationic state. This shortcoming was partially alleviated by introducing the electrostatic potential (ϕ) as another component of the optimization loss function (eq 1). As a result, the optimized partial charges reproduce the distribution of the electrostatic potential around the model compound with reasonable accuracy (RMSD of 3 to 5 units of *k*_*B*_ *T*). Note that a slightly better agreement between the QM and MM electrostatic potentials can be achieved by increasing the weight of this component in the optimization loss function. However, that would impair the description of the water–compound interactions, which were the main objective of the optimization and are key constituents of the original CHARMM optimization scheme.^32^ In summary, the optimized partial charges demonstrate a good balance between reproducing the interactions of the TRP and TYR side chains with water, while also describing well their general electrostatic properties.

### Optimization of Bond, Angle, and Stiff-Dihedral Parameters

In the next step, we performed PES for all bonds and angles in the model compounds, to see how their energetics change upon TYR and TRP oxidation. Figures 2 and 3 show the PES for bonds and angles that demonstrate the most pronounced change in energetics in 3MI and 4MP, respectively. In 3MI, bonds between heavy atoms demonstrate a prominent change in their energetics, while bonds involving hydrogen atoms are largely unaffected (Figures S2 and S3). Interestingly, the oxidation of 3MI changes the energetics of the bonds similarly in both the neutral and cationic radical states (Figure 2A). In contrast, the energetics associated with the deformations of angles remain essentially intact during the transition from the nonradical to the cationic radical state, but demonstrate a notable change during the transition to the neutral radical state (Figure 2B). Thus, it is the electron configuration that affects the energetics and geometry of the bonds, and it is the deprotonation that changes the energetics and geometry of the angles. Similar trends were observed in 4MP (Figures S4 and S5), except for the hydroxyl CZ–OH bond, which is significantly more contracted in the neutral radical state compared to the cationic radical state (Figure 3).

**Figure 2:**
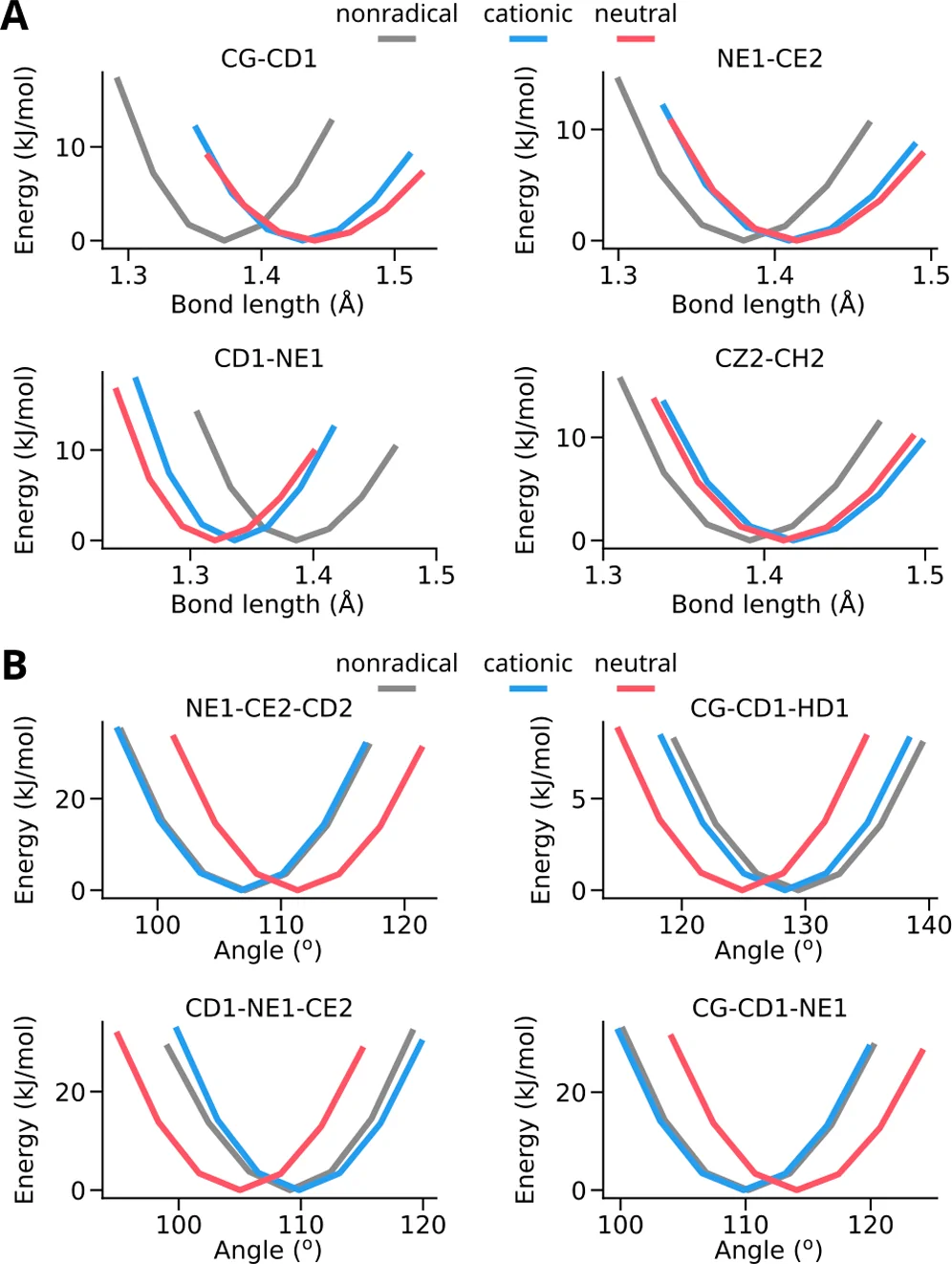
Potential energy scans for bonds and angles in 3-methylindole conducted at the QM level of theory. **A**, **B** Potential energy scans for bonds (**A**) and angles (**B**) that demonstrate the largest differences between the nonradical and radical states.

**Figure 3:**
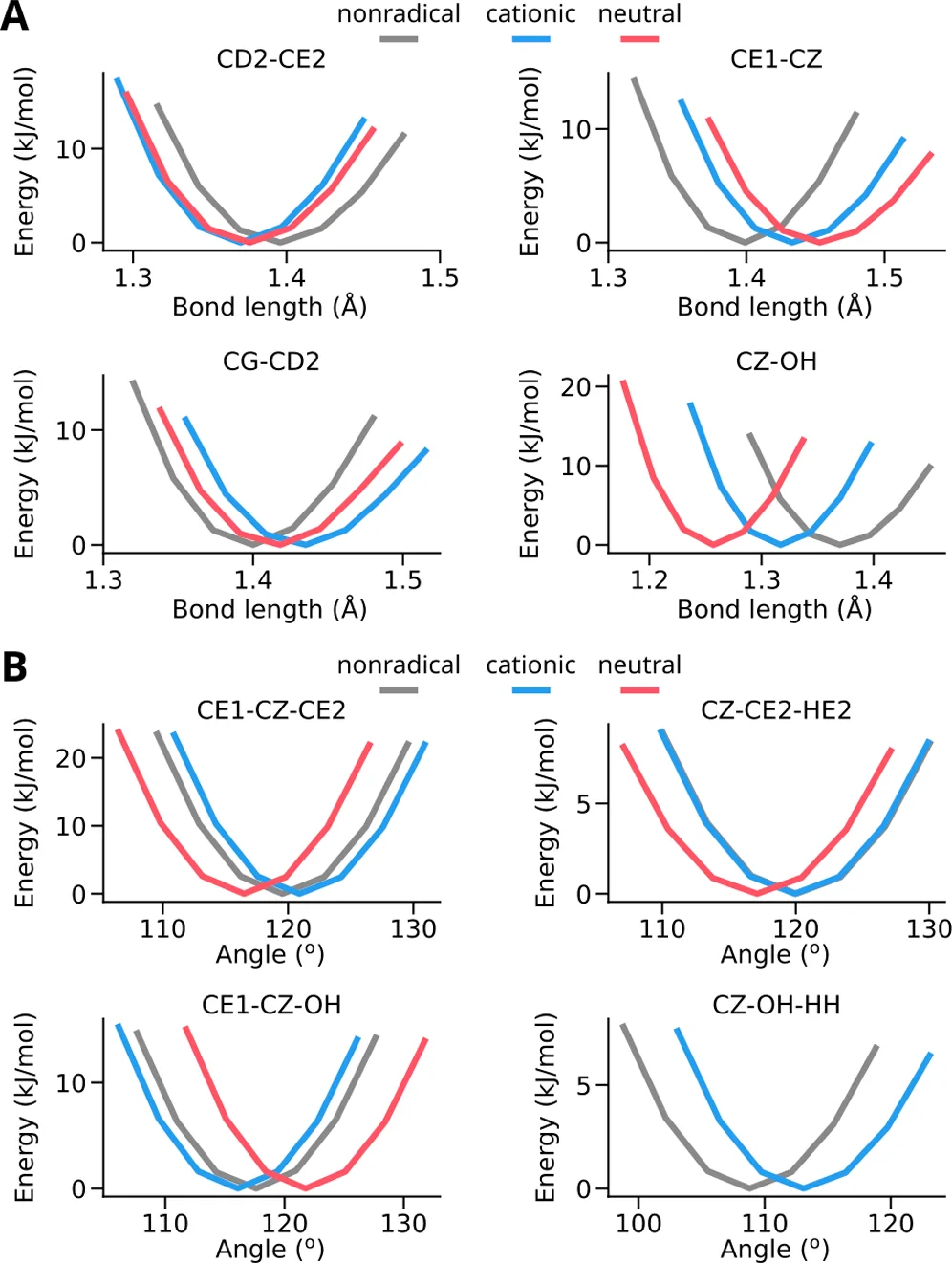
Potential energy scans for bonds and angles in 4-methylphenol conducted at the QM level of theory. **A**, **B** Potential energy scans for bonds (**A**) and angles (**B**) that demonstrate the largest differences between the nonradical and radical states.

In the original CHARMM parametrization scheme, bond and angle parameters were developed based on QM calculations at the MP2 level of theory.^32^ In contrast, we employed density functional theory (DFT) with the B3LYP functional, because of its higher computational efficiency and the lack of spin contamination, which requires special attention in MP2 calculations on radical species.^67^ To demonstrate that DFT and MP2 provide comparable results, we performed PES at the MP2 level of theory for bonds and angles that demonstrated the most notable differences in their energetics between the nonradical and cationic radical states of 3MI. Figure S6 shows that the difference in PES between the MP2 and DFT methods is negligible compared to the interstate differences, justifying further usage of DFT with the B3LYP functional in the parametrization procedure.

To account for the change in the intramolecular energetics in the radical states, we parametrized the selected bonded terms in the model compounds. The parametrization required the introduction of new atom types, that were specific to the radical states. New atom types were assigned only to atoms that were involved in bonded interactions whose energetics were sensitive to the change in the oxidation state of the parametrized compounds. In particular, new state-specific atom types were introduced into the force field for the NE1, CG, and CZ2 atoms in 3MI, and for the CG, CE1, CE2, CZ, and OH atoms in 4MP. Most of these atoms correspond to regions of high spin density in 3MI and 4MP in the radical states as shown in Figure 1. The parameters for the bonds and angles involving the new atom types were optimized to match the PES calculated at the MM and QM levels of theory. The equilibrium values and the force constants of bonds and angles were subject to optimization, resulting in a set of parameters that performed well against the QM data (Figures S7–S10).

Finally, we also optimized parameters for stiff dihedral degrees of freedom that are omnipresent in the side chains of TYR and TRP, stabilizing their planar structures. The change in the oxidation state of 3MI results in the softening of some dihedrals, while others become stiffer (Figures S11 and S12). Both of these cases are described well through the optimized parameters (Table 2). In turn, the stiff dihedrals in 4MP are well described by the original set of CHARMM parameters and required nearly no additional optimization (Figures S13 and S14). We also optimized dihedral parameters that describe the rotation of the hydroxyl group in the cationic radical state of 4MP. Interestingly, the hydroxyl dihedral becomes much stiffer compared to the standard state, virtually prohibiting transitions between the two energy minima (Figure S15). This result suggests that the hydroxyl group of TYR would be trapped in the orientation it acquired just before electron extraction, affecting the mechanism of the following deprotonation. The effect, however, would only be relevant if TYR is stabilized by its environment in its cationic radical state which is highly unstable otherwise.^68^

**Table 2:** Optimization of Bond, Angle, and Stiff-Dihedral Parameters^a^

	3-methylindole radical		4-methylphenol radical	
	neutral	cationic	neutral	cationic
bonds (kJ/mol)	0.42/1.19	0.33/0.75	0.52/1.48	0.73/1.91
angles (kJ/mol)	0.81/1.97	0.73/1.90	0.68/1.69	0.62/1.84
dihedrals (kJ/mol)	0.51/1.41	0.49/1.54	0.64/1.81	0.77/2.02

a Data are shown as RMSD/Max |err|, where RMSD is the root-mean-square deviation and Max |err| is the maximum absolute error in reproducing PES for bonds, angles, and stiff dihedrals calculated at the QM level of theory by the optimized bonded parameters.

Table 2 summarizes the performance of the optimized MM parameters against the QM data. The PES conducted with the final set of MM parameters have RMSD values well below 1 kJ/mol and maximum absolute errors below *k*_*B*_ *T* when compared to the PES conducted at the QM level of theory. Figure 4 additionally shows PES for bonds, angles, and stiff dihedrals in 3MI with the highest RMSD between the MM and QM data, demonstrating the high quality of the optimized MM parameters and an improvement over the unadjusted standard CHARMM parameters.

**Figure 4:**
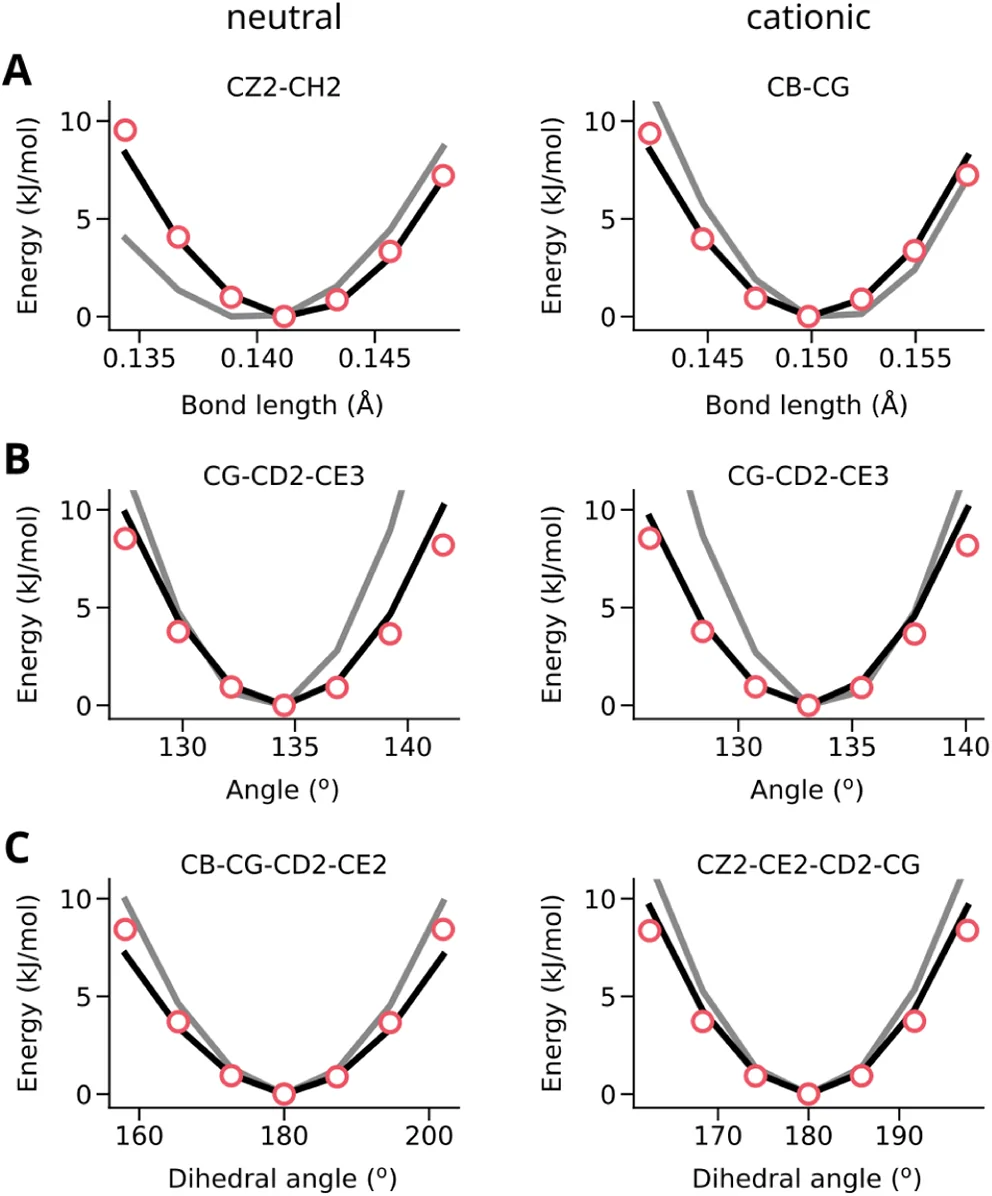
Optimization of bond, angle, and stiff-dihedral parameters in 3-methylindole. **A**, **B**, **C** MM potential energy scans for bonds (**A**), angles (**B**), and stiff dihedrals (**C**) obtained with the initial (gray) or optimized (black) MM parameters with the highest RMSD with respect to the QM data. Shown are the scans for the neutral (left) and cationic (right) radical states of 3MI. QM data are shown as red circles.

### Optimization of Soft-Dihedral Parameters

Soft dihedrals are characterized by an energy landscape that is less steep compared to the stiff dihedrals, allowing molecular rotation around saturated bonds. As soft-dihedral degrees of freedom play a crucial role in the conformational dynamics of proteins, their parametrization is of particular importance for the description of protein conformational changes in MD simulations. Whereas stiff dihedrals preserve the planar structure of the phenol and indole rings in the TYR and TRP residues, the soft-dihedral angles χ_1_ and χ_2_ define their side-chain rotation. To optimize parameters that describe the TYR and TRP side-chain rotation in the radical states, we performed rotational PES along the χ_1_ and χ_2_ angles using dipeptide models (Figures 5, 6, 7, S16). Parametrization of the side-chain dihedrals requires that the remaining dihedral angles remain constrained. In particular, changes in the backbone dihedral angles during the PES can interfere with the parametrization. Therefore, the dipeptide backbone is commonly fixed to an arbitrary (usually α-helical) conformation during amino acid parametrization. To ensure a proper description of side-chain rotation in various secondary-structure contexts, we performed the PES for four different backbone conformations. In addition to the α-helical conformation (*alpha*), we also considered backbone conformations of the β-strand (*beta*), left-handed α-helix (*l-handed*), and collagen helix (*collagen*). All these conformations occur in the model protein systems that are used to study PCET^15,50^ as well as in natural proteins whose functions rely on the oxidation of the TYR and TRP residues.^26,33^ All PES for all the backbone conformations were simultaneously targeted during the parameter optimization.

**Figure 5:**
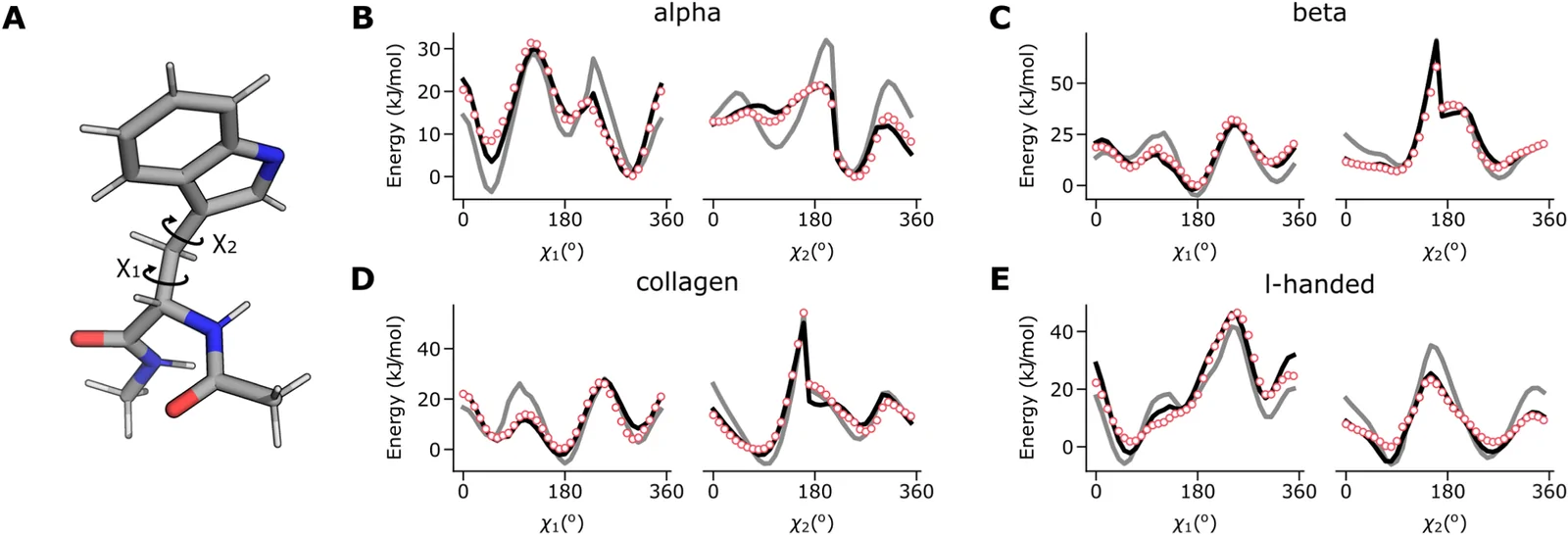
Optimization of soft-dihedral parameters for the TRP dipeptide in the neutral radical state. **A** TRP dipeptide in the neutral radical state with the indicated angles χ_1_ and χ_2_. **B**, **C**, **D**, **E** MM potential energy scans along the χ_1_ and χ_2_ dihedral angles with the dipeptide backbone restrained to the *alpha* (**B**), *beta* (**C**), *collagen* (**D**), or *l-handed* (**E**) conformation. Shown are the scans conducted with the unadjusted (gray) and optimized (black) MM parameters. QM data are shown as red circles.

**Figure 6:**
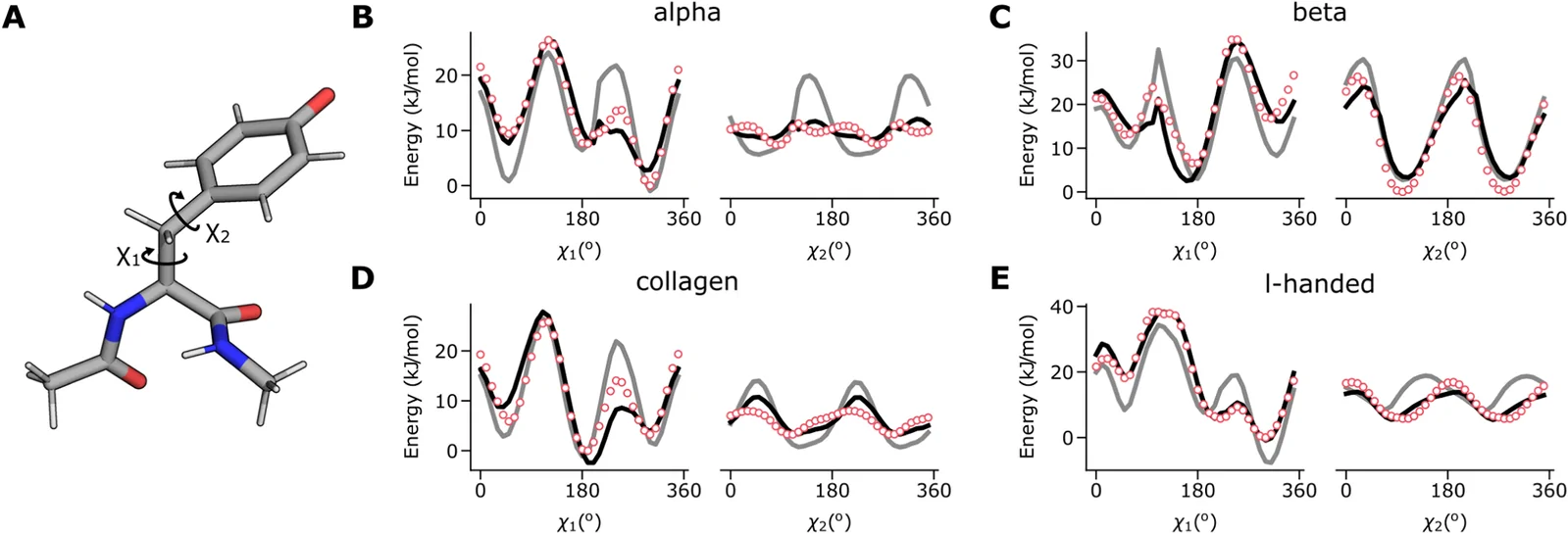
Optimization of soft-dihedral parameters for the TYR dipeptide in the neutral radical state. **A** TYR dipeptide in the neutral radical state with the indicated angles χ_1_ and χ_2_. **B**, **C**, **D**, **E** MM potential energy scans along the χ_1_ and χ_2_ dihedral angles with the dipeptide backbone restrained to the *alpha* (**B**), *beta* (**C**), *collagen* (**D**), or *l-handed* (**E**) conformation. Shown are the scans conducted with the unadjusted (gray) and optimized (black) MM parameters. QM data are shown as red circles.

**Figure 7:**
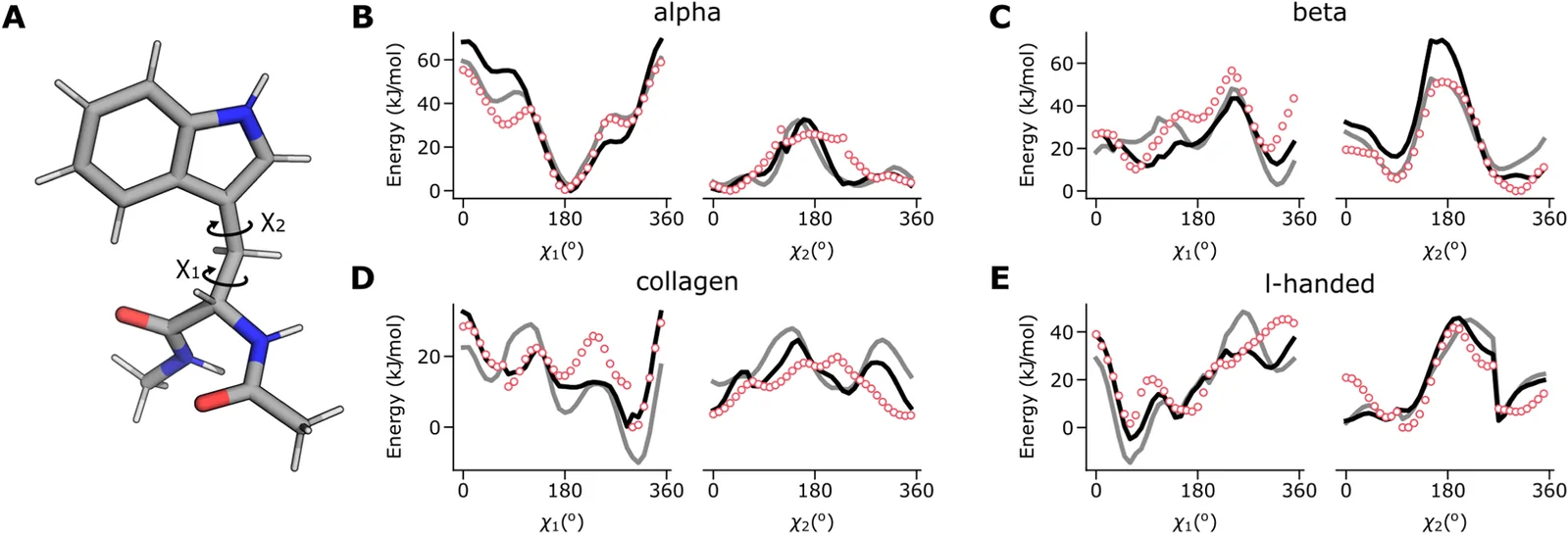
Optimization of soft-dihedral parameters for the TRP dipeptide in the cationic radical state. **A** TRP dipeptide in the cationic radical state with the indicated angles χ_1_ and χ_2_. **B**, **C**, **D**, **E** MM potential energy scans along the χ_1_ and χ_2_ dihedral angles with the dipeptide backbone restrained to the *alpha* (**B**), *beta* (**C**), *collagen* (**D**), or *l-handed* (**E**) conformation. Shown are the scans conducted with the unadjusted (gray) and optimized (black) MM parameters. QM data are shown as red circles.

Figure 5 shows the PES obtained at the MM and QM levels of theory, calculated for different backbone conformations of the TRP dipeptide in the neutral radical state. The optimized MM parameters reproduce well the positions and the relative energies of the local minima, and the potential energy barriers observed in the reference QM data. Overall, the optimized parameters for the TRP residue in the neutral radical state demonstrate an improvement over the unadjusted set of parameters, reducing by half the RMSD between the MM and QM PES (Table 3). The dihedral parameters for the TYR residue in the neutral radical state perform similarly well (Figure 6), with the overall RMSD between the MM and QM PES of around *k*_*B*_ *T* (Table 3). In particular, the optimized parameters properly capture the flattening of the energy landscape along the χ_2_ dihedral angle with the backbone in the α-helical conformation (Figure 6B), that is expected to have a prominent impact on the conformational dynamics of the oxidized TYR residue in protein systems. The optimized parameters for TRP in the cationic radical state demonstrate a more moderate improvement in the description of the side-chain rotation (Table 3). The parameters describe well the energetics of the χ_1_ dihedral angle, properly capturing the relative energies and the positions of the local minima (Figure 7). However, the energetics of the χ_2_ dihedral angle is described less precisely, although general features such as the energy barrier near 180° are reproduced in most of the PES.

**Table 3:** Optimization of Soft-Dihedral Parameters^a^

	TRP radical		TYR radical	
	neutral	cationic	neutral	cationic
alpha	2.0/6.2	9.1/7.3	1.7/5.2	8.8/5.1
beta	2.9/5.7	9.1/10.0	3.6/4.4	7.4/10.6
collagen	2.8/5.5	5.5/10.0	2.7/3.7	5.3/9.1
l-handed	3.0/6.7	7.0/10.4	1.9/6.4	8.4/13.2
overall	2.7/6.0	7.7/9.5	2.6/5.0	7.5/9.6

a Data are shown as RMSD*^opt^*/RMSD*^init^* (kJ/mol), where RMSD is the root-mean-square deviation between PES along the soft-dihedral angles conducted at the QM and MM levels of theory. The RMSD was calculated for the PES obtained with the optimized (RMSD*^opt^*) and unadjusted nonradical CHARMM parameters (RMSD*^init^*).

In the cationic radical state of TYR, optimization of the side-chain dihedral parameters also resulted in an overall better agreement with the QM data in terms of RMSD as compared to the unadjusted CHARMM parameters (Table 3). However, the optimized parameters were less efficient in capturing certain details of the energetics of the side-chain rotation. In particular, both the unadjusted and optimized MM parameters fail to properly describe side-chain rotation along the χ_2_ and χ_1_ dihedral angles with the backbone in the *collagen* and *l-handed* conformations, respectively (Figure S16). Since partial charges directly affect the energetics of dihedral rotation, we estimated the atomic partial charges of the dipeptides using the Minimal Basis Iterative Stockholder method^69^ for all conformations considered in the dihedral PES (Figure S17). We found that the dipeptide backbone carries a substantial positive charge in the cationic radical states, with the effect being particularly pronounced for the cationic radical state of TYR. In contrast, the backbone remains neutral in the neutral radical states, suggesting that backbone polarization might contribute to the difficulty of optimizing the dihedral parameters for the cationic radical states. The issue could potentially be mitigated by optimizing the partial charges using dipeptide models to account for backbone polarization. However, the broad distribution of the backbone charge across the dipeptide conformations in the cationic radical states (Figure S17) suggests that any fixed set of partial charges would interfere with the optimization of the dihedral parameters within the fixed-charge MM framework. For the sake of completeness, we still provide the parameters for the side-chain dihedrals of the TYR residue in the cationic radical state, although we note that results from any long-timescale MD simulations of proteins with TYR in that specific redox state should be interpreted with great caution. Except for the TYR residue in the cationic radical state, however, the resulting set of parameters demonstrates a clear improvement over the unadjusted CHARMM parameters in describing the energetics of the side-chain rotation of TYR and TRP radicals, with a particularly good description of the neutral radical states.

### Testing Simulations

#### Peptide A

We conducted a series of MD simulations of model protein systems, to demonstrate and validate the applicability of the force field parameters presented here for studying the effects of TYR and TRP oxidation on the conformational dynamics and biophysical properties of proteins. The optimized parameters for the TYR residue in the neutral radical state were tested in simulations of peptide A, a β-hairpin maquette protein that is used to study ET and PCET.^15^ Peptide A has a single TYR residue (Y5) that can be used to specifically study the effects of TYR oxidation on the protein structure and dynamics. Peptide A was simulated with Y5 in the nonradical and neutral radical states, to study the effects of TYR oxidation on its structural properties. In the nonradical state, the peptide demonstrates high stability preserving its initial β-hairpin structure (Figure 8A, Figure S18A). In contrast, we observed a decrease in the overall structuring of the peptide when Y5 was in the neutral radical state (Figure S18B). However, the peptide does not completely lose its secondary structure, with a substantial fraction of residues retaining the β-strand conformation (Figure 8A,B). Thus, the new set of force field parameters results in good agreement with the available experimental data which suggest a partial loss of hydrogen bonds in peptide A upon oxidation of Y5.^70^

**Figure 8:**
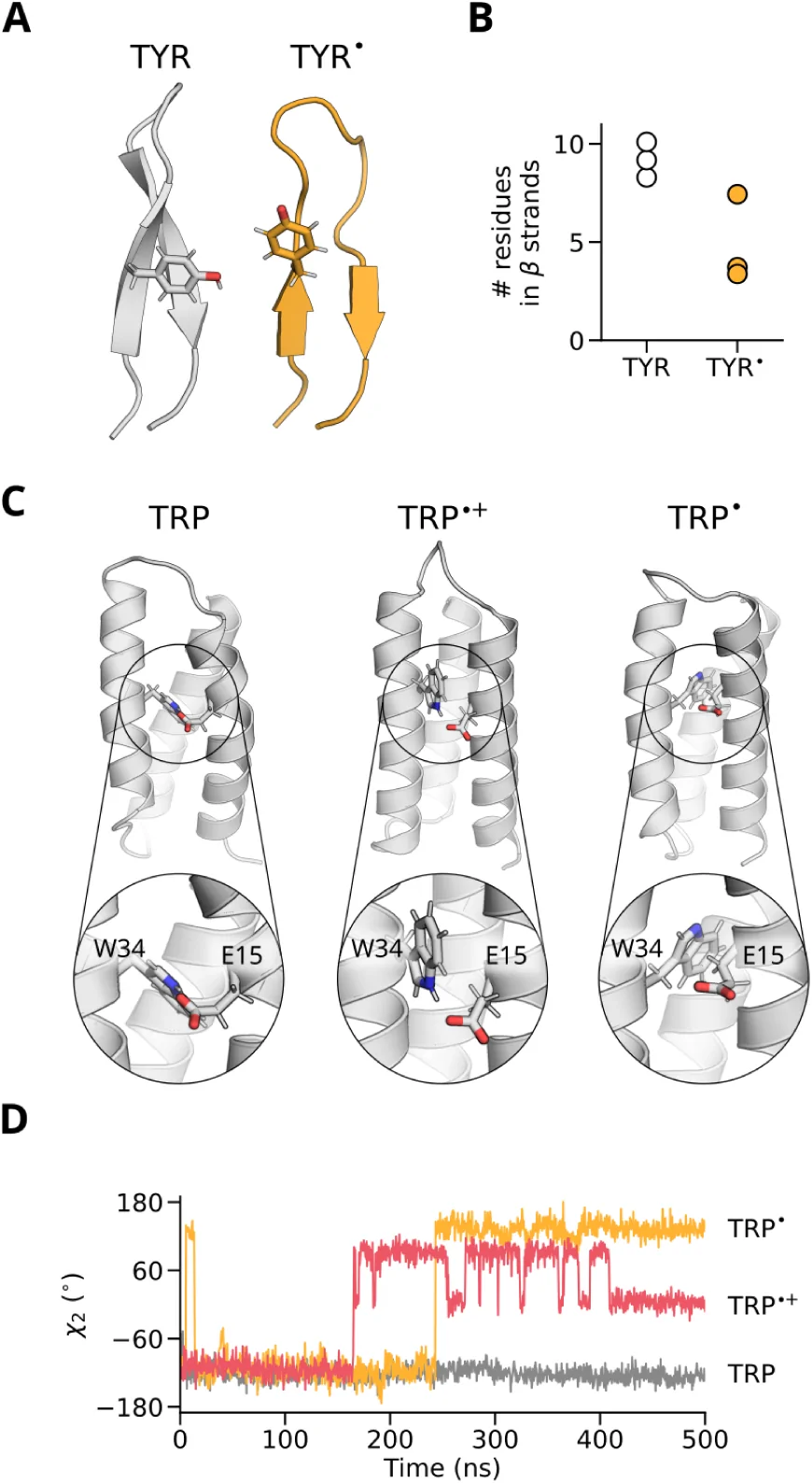
Testing new parameters in model systems. **A** Structure of peptide A after a 1-μs MD simulation with Y5 in the nonradical and neutral radical states. **B** Average number of residues in the β strands in the simulations of peptide A with Y5 in the nonradical and neutral radical states. Each data point corresponds to an independent simulation replica. **C** Structure of α_3_W after 500 ns of simulations with W34 in the nonradical, cationic radical, and neutral radical states. Residues W34 and E15 are shown as sticks. **D** Time course of the χ_2_ angle of W34 in simulations of α_3_W with W34 in the nonradical, cationic radical, and neutral radical states.

#### Radical Maquette α_3_W

We tested the new force field parameters for the TRP residue in simulations of a model protein α_3_W, which contains a single TRP residue (W34) and is often used to study PCET in proteins.^16,19,50^ In the nonradical state, W34 forms a rather stable bond with E15, as illustrated in Figure 8C. Although this bond can be preserved in the cationic radical state, its geometry is drastically changed (Figure 8C) because of the pronounced rotation of W34 along the χ_2_ dihedral angle (Figure 8D). As a result, a notable change in the overall fold of α_3_W is observed (Figure S19). To quantify the effect of the change in the total charge of W34 on the protein electrostatics, we calculated the electrostatic potential sensed by GLU and LYS residues that abundantly cover the surface of α_3_W (Figure S20). The analysis revealed only a minor change in the electrostatic potential with no clear trend in all the residues except for K38, which is located one-helical-turn down from W34. The electrostatic potential of other residues that are located close to W34 remains largely unchanged, suggesting that it is not the positive charge of W34 that increases the electrostatic potential of K38. Instead, the reorientation of W34 seems to disrupt the efficient screening of K38 by the neighboring acidic residues. In particular, K38 forms 3.4 ± 0.2 and 1.6 ± 0.1 contacts with GLU residues when W34 is modeled in the nonradical and cationic radical states, respectively.

Notably, simulating W34 in the neutral radical state preserves the original fold of α_3_W (Figure S19), although W34 can experience a pronounced rotation along the χ_2_ dihedral angle (Figure 8D). As deprotonation of W34 strongly disfavors formation of a bond with E15, the former can bury its less hydrophilic side chain into the protein interior (Figure 8C,D), leading to a notable reduction in its solvent-accessible surface area that drops from 11 Å^2^ to 3 Å^2^. Such an orientation of W34 corresponds to a local minimum in the PES along the χ_2_ angle (Figure 5B), which is separated from the global minimum by an energy barrier. This barrier is significantly higher in the nonradical state of TRP and would prevent efficient interconversion between rotamers, demonstrating the importance of a proper description of dihedral energetics in the radical states of the TRP residues. Thus, our simulations reveal that oxidation of TRP can lead to a pronounced change in the side-chain orientation and in the overall fold of proteins, additionally affecting their electrostatic properties.

#### Pigeon Cryptochrome 4

Radical states of TRP play a crucial role in avian magnetoreception that is based on the magnetic sensitivity of the radical pair formed between flavin adenine dinucleotide (FAD) and a TRP residue inside a cryptochrome photoreceptor protein.^8,9,61,71^ The radical pair is formed as a result of FAD photoexcitation that is followed by an ET process along the chain of TRP residues (Figure 9A), resulting in the anionic semiquinone state of FAD and the cationic radical state of a TRP residue.^14,72,73^ Residues W318 (TrpC) and W369 (TrpD) located at the end of the ET chain play particularly important roles in the magnetoreception mechanism.^14,73^ As the dynamics of TrpC and TrpD have been previously shown to affect the magnetic sensitivity of cryptochromes^61,71^ we tested the new force field parameters in the simulations of a pigeon cryptochrome (ClCry4), with a particular focus on the fluctuations in the librational and dihedral angles of the TrpC and TrpD residues. The simulations were conducted with ClCry4 in the radical-pair state with the FAD cofactor in the anionic semiquinone state and either TrpC or TrpD in the cationic radical state. The librational motions of the TRP residues were quantified by analyzing fluctuations of the principal axes of their indole groups (Figure 9B). Figure 9C shows the distribution of the librational angles of TrpC and TrpD in the cationic radical state. The data were fitted with the Rayleigh distribution and used its scaling parameter to compare librational motions of TRP residues in different redox states (Table S1). Similar to the previous study,^61^ TrpD demonstrates more pronounced fluctuations than TrpC. However, the scaling parameter is systematically higher than previously reported^61^ in both TrpC and TrpD for all the principal axes. Although the observed difference of ∼0.8° is unlikely to affect the magnetic sensitivity of the radical pair, the results indicate higher flexibility of the indole ring in simulations with the optimized bonded parameters. We also analyzed fluctuations in the side-chain dihedral angle Λ of TrpC and TrpD (Figure S21). The resulting distributions of the Λ angle are very similar to those reported previously,^61^ with average angle values of −79.5° and 75.6° in TrpC and TrpD, respectively. The earlier simulations of ClCry4 were conducted using the bonded parameters of the nonradical state of TRP to describe TrpC and TrpD in the cationic radical state. Therefore, we conclude that the sub-μs dynamics of the side chains of the TrpC and TrpD residues in the cationic radical state are mainly defined by the protein environment and are not very sensitive to changes in the energetics of the bonded interactions arising from oxidation.

**Figure 9:**
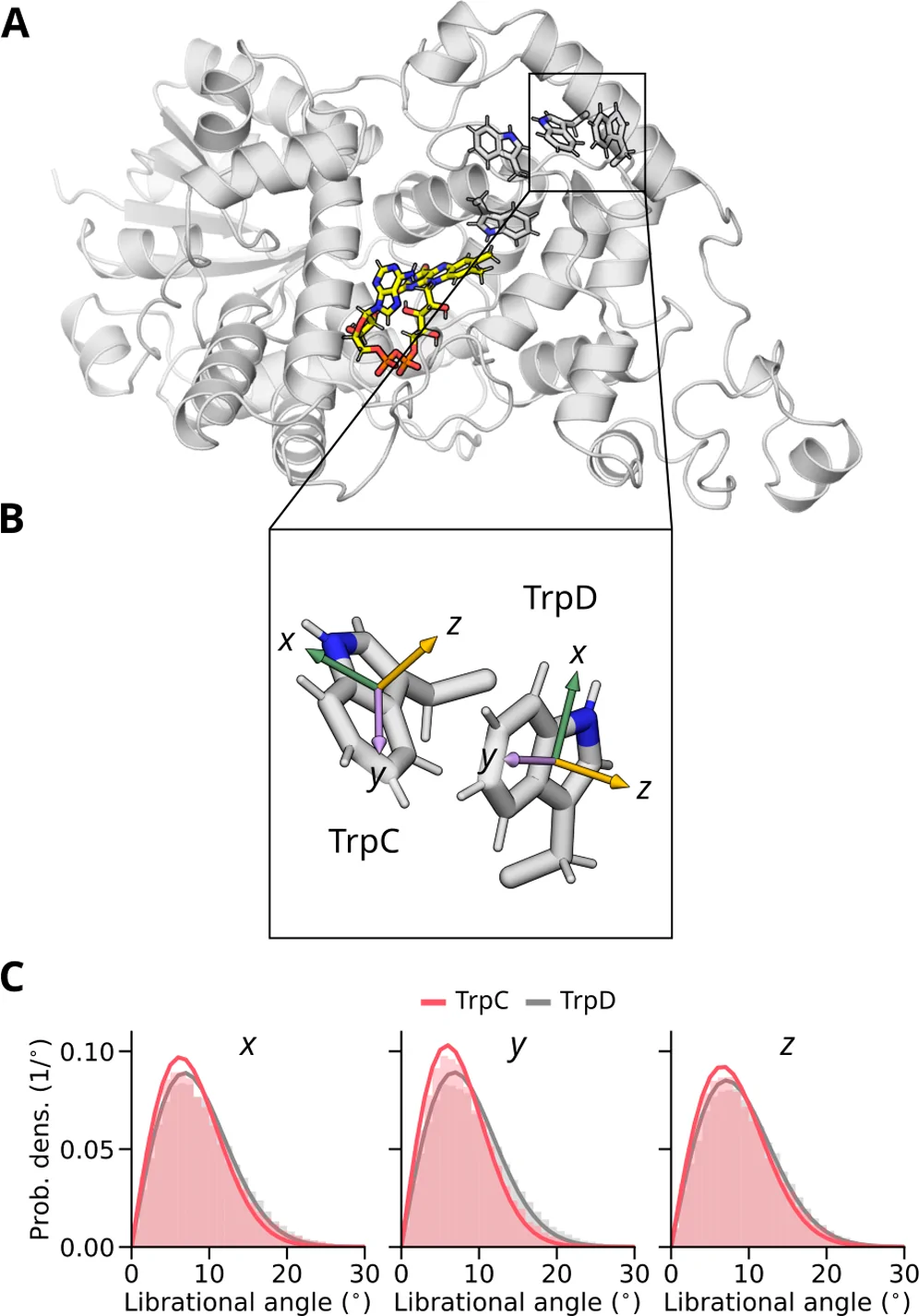
Librational motions of TRP in ClCry4. **A** Structure of ClCry4 (PDB ID: 6PU0) with the FAD cofactor and the conserved ET chain of TRP residues shown in stick representation. **B** Orientation of the principal axes that were used to quantify the librational motion of TrpC and TrpD. **C** Probability-density distributions of the librational angles around the indicated axes obtained from the MD simulations of ClCry4 with TrpC (red) or TrpD (gray) in the cationic radical state. Fits to the underlying histograms with the Rayleigh distribution are shown by solid lines. Each distribution corresponds to the cumulative data from three independent simulation replicas.

## Conclusions

All-atom MD simulations allow for studying effects of amino acid oxidation on the conformational dynamics and biophysical properties of proteins that have redox-active residues, such as TYR and TRP. To connect changes in the protein dynamics with changes in the redox states of TYR and TRP by means of MD simulations, these states have to be parametrized within the MM framework. Here, we present a set of optimized force field parameters for the radical states of the TYR and TRP residues. The parameters were optimized against a large body of QM data on the intramolecular energetics, electrostatic properties, and interactions of the TYR and TRP residues with water. Particular attention was paid to the parametrization of the side-chain dihedrals, ensuring a proper description of the side-chain dynamics in various secondary structure contexts. The resulting parameters show good agreement with the reference QM data and demonstrate the necessity to account for changes in the intramolecular energetics of oxidized amino acid residues to study them within the MM framework.

Our benchmark simulations demonstrate the validity and applicability of the new set of parameters to study the effects of TYR and TRP oxidation on protein dynamics, ranging from side-chain rotation to global conformational changes. In particular, oxidation of the TYR and TRP residues can lead to destabilization of the secondary structure and to an overall change in the tertiary structure of proteins (Figure 8). The observed conformational changes demonstrate the applicability of the presented force field parameters for studying the roles of TYR and TRP radicals in biomolecular signaling using exploratory MD simulations. In turn, oxidation-induced deprotonation can lead to the side-chain reorientation caused by changes in the hydrophilicity and side-chain energetics of the residue (Figure 8). Our simulations suggest that such reorientation can result in a prominent change in the residue’s hydration with potential implications for PCET mechanisms.^19,21^ Finally, the simulation results demonstrate the applicability of the presented parameters to study the effects of TRP dynamics in the radical states on the mechanism of magnetoreception^61,71^ (Figure 9). In conclusion, the presented set of force field parameters paves the way to study the biological roles of the TYR and TRP radicals in various biomolecular systems at atomistic resolution using MD simulations.

## Supplementary Material





## Data Availability

The developed force field parameters are available in the Supporting Information files. ORCA (version 5) and GROMACS (version 2019 and 2025) software packages are available at the official websites (https://orcaforum.kofo.mpg.de and https://www.gromacs.org). *g*_*elpot* tool is available at https://jugit.fz-juelich.de/computational-neurophysiology/g_elpot.

## Notes

Archive containing developed force field parameters in the GROMACS and NAMD formats (ZIP)
Water–compound configurations used for charge optimization; all potential energy scans for bonded degrees of freedom; backbone charge in the dipeptide models; secondary-structure assignment in the peptide A simulations; data on the structural stability of α_3_W; electrostatic potential of the GLU and LYS residues of α_3_W; data on the fluctuations of the librational and dihedral angles of TRP residues in ClCry4 (PDF)
