# Supplementary material for: Developing CHARMM Force Field Parameters for the Radical States of Tryptophan and Tyrosine
Source: J Chem Theory Comput. 2026 Sep 1;22(18):9790–801. doi: 10.1021/acs.jctc.6c01280 (PMC13602919; doi:10.1021/acs.jctc.6c01280)
Supplement: Supplementary file 1 [file ct6c01280_si_002.pdf]

Supporting Information

Developing CHARMM force-field parameters for the radical states of tryptophan and tyrosine

Andrei Y. Kostritski,[*,†] Jonathan Hungerland,[†] Christian Schuster,[†] and Ilia A. Solov'yov[†,‡,¶]

†*Institute of Physics, School of Mathematics and Science, Carl von Ossietzky Universität Oldenburg, Carl-von-Ossietzky Str. 9-11, Oldenburg 26111, Germany*

‡*Research Center for Neurosensory Sciences, Carl von Ossietzky Universität Oldenburg, Carl-von-Ossietzky Str. 9-11, Oldenburg 26111, Germany*

¶*Institute of Physics, Center for Nanoscale Dynamics (CENAD), Carl von Ossietzky Universität Oldenburg, Oldenburg 26129, Germany*

E-mail: andrei.kostritskii@uni-oldenburg.de

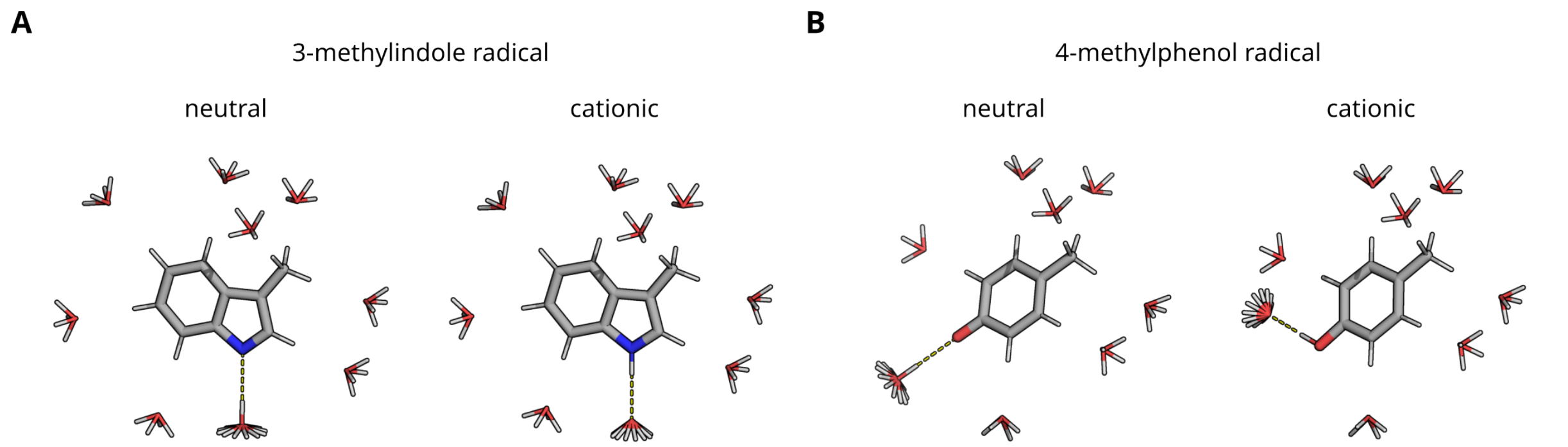

Figure S1: **Water-compound configurations used for charge optimization in the model compounds.** Shown are configurations to parametrize 3-methylindole (**A**) and 4-methylphenol (**B**) as model compounds for TRP and TYR, respectively. Note that each configuration contained only a single water molecule. The dashed line illustrates the distance between the oxygen of the water molecule and the hydrogen-bond donor/acceptor of the model compound.

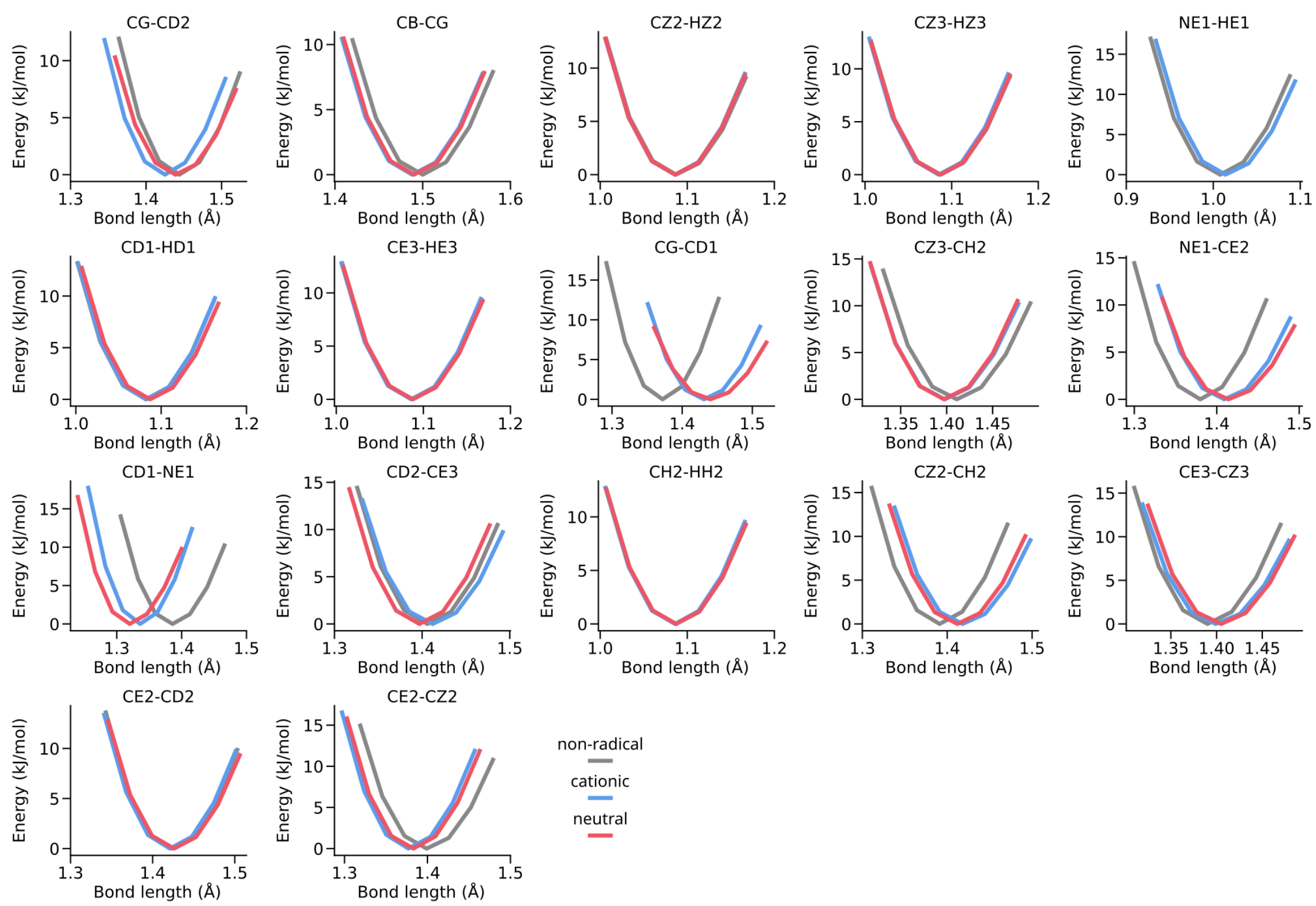

Figure S2: **Potential energy scans (PES) for bonds in 3-methylindole conducted at the QM level of theory.** Shown are PES conducted for the non-radical (gray), neutral radical (red) and cationic radical (blue) states.

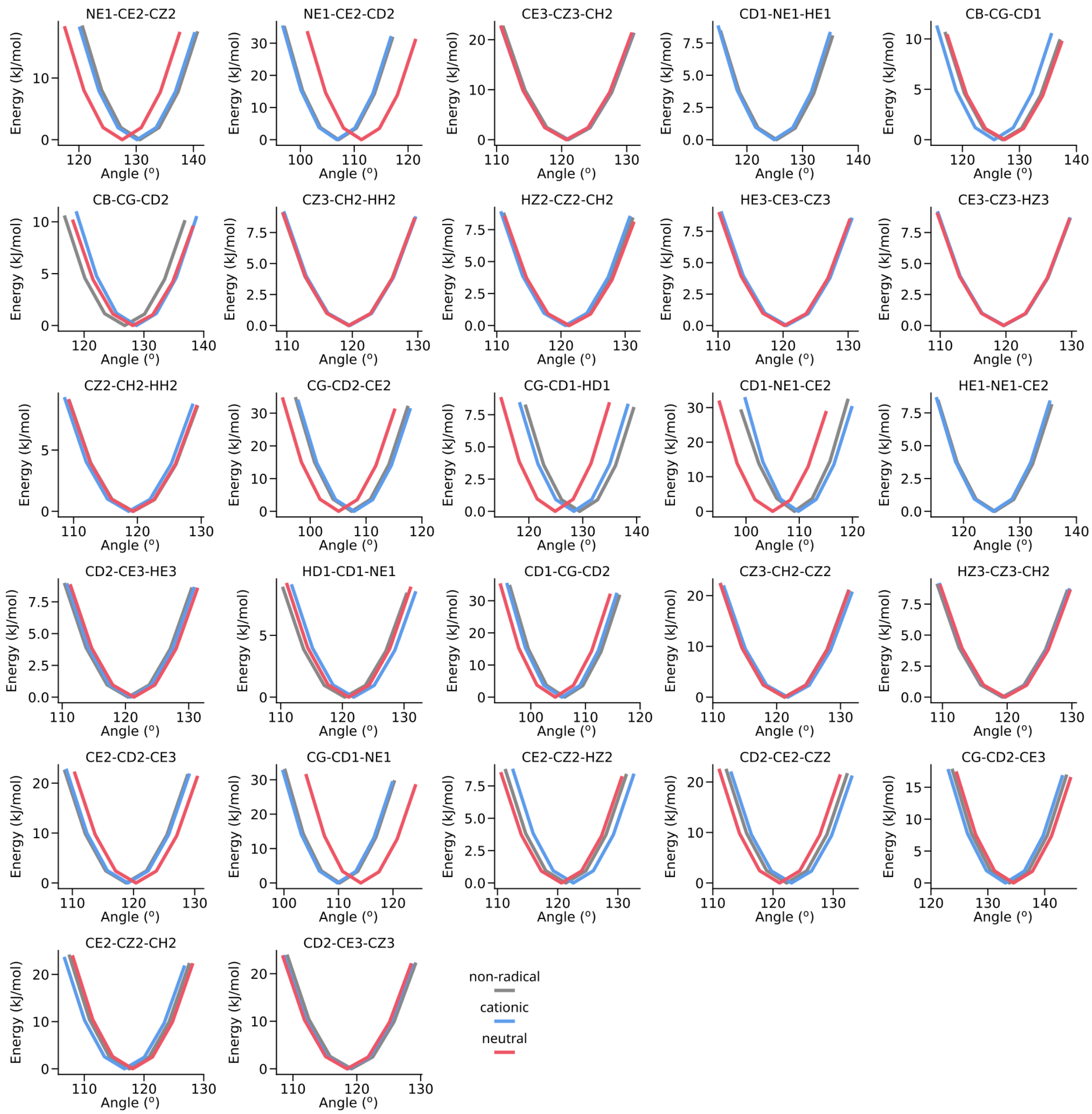

Figure S3: **Potential energy scans (PES) for angles in 3-methylindole conducted at the QM level of theory.** Shown are PES conducted for the non-radical (gray), neutral radical (red) and cationic radical (blue) states.

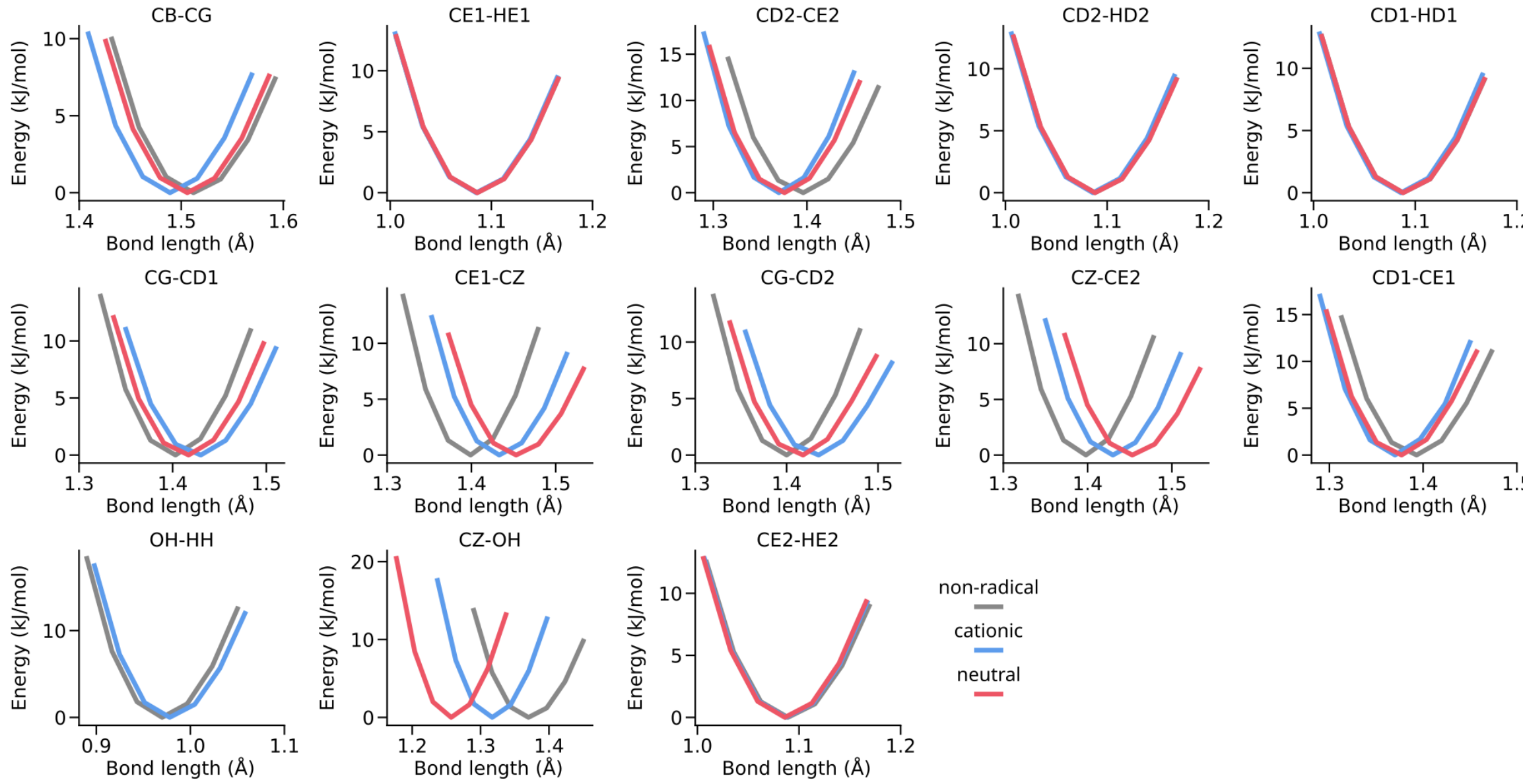

Figure S4: **Potential energy scans (PES) for bonds in 4-methylphenol conducted at the QM level of theory.** Shown are PES conducted for the non-radical (gray), neutral radical (red) and cationic radical (blue) states.

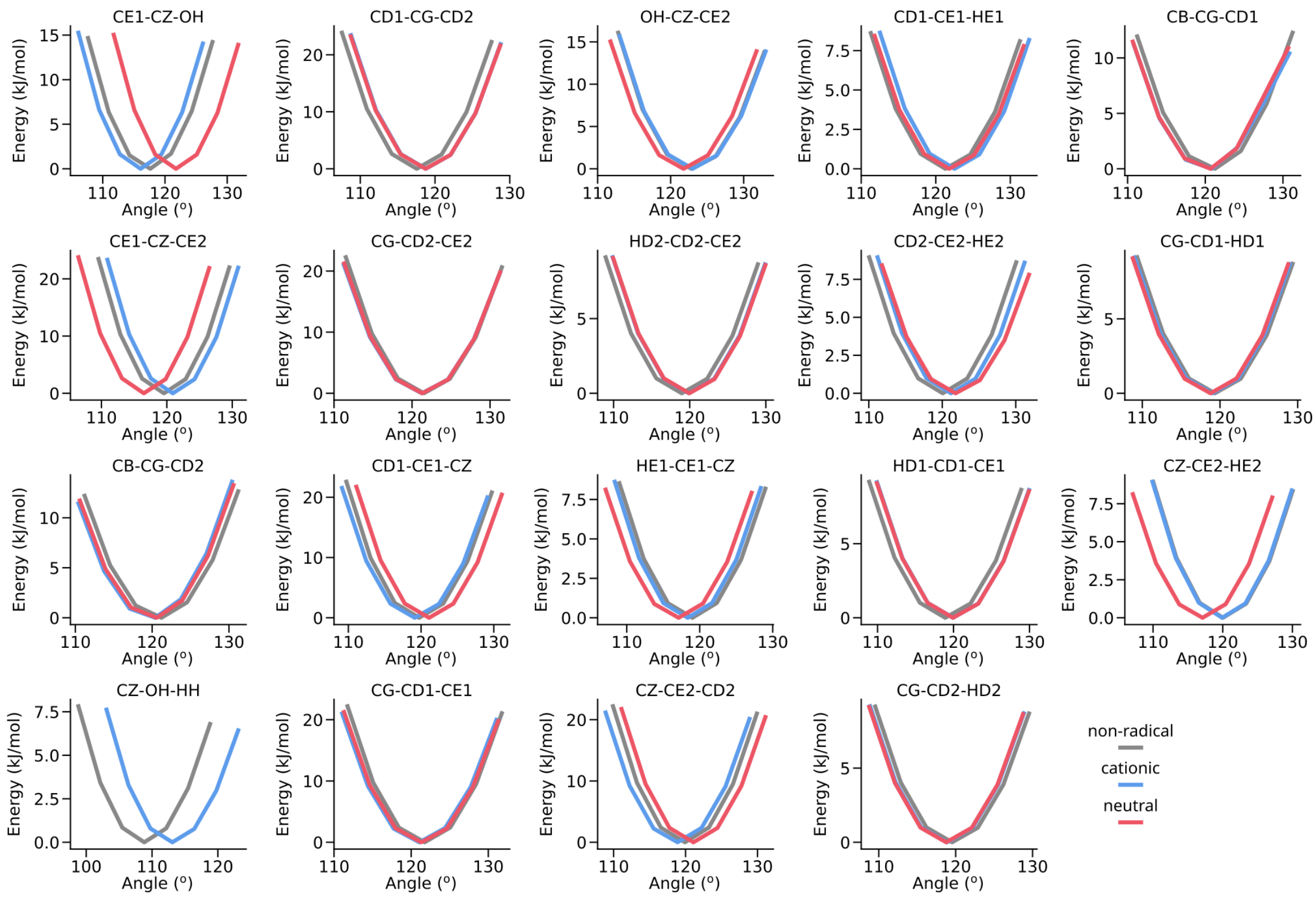

Figure S5: **Potential energy scans (PES) for angles in 4-methylphenol conducted at the QM level of theory.** Shown are PES conducted for the non-radical (gray), neutral radical (red) and cationic radical (blue) states.

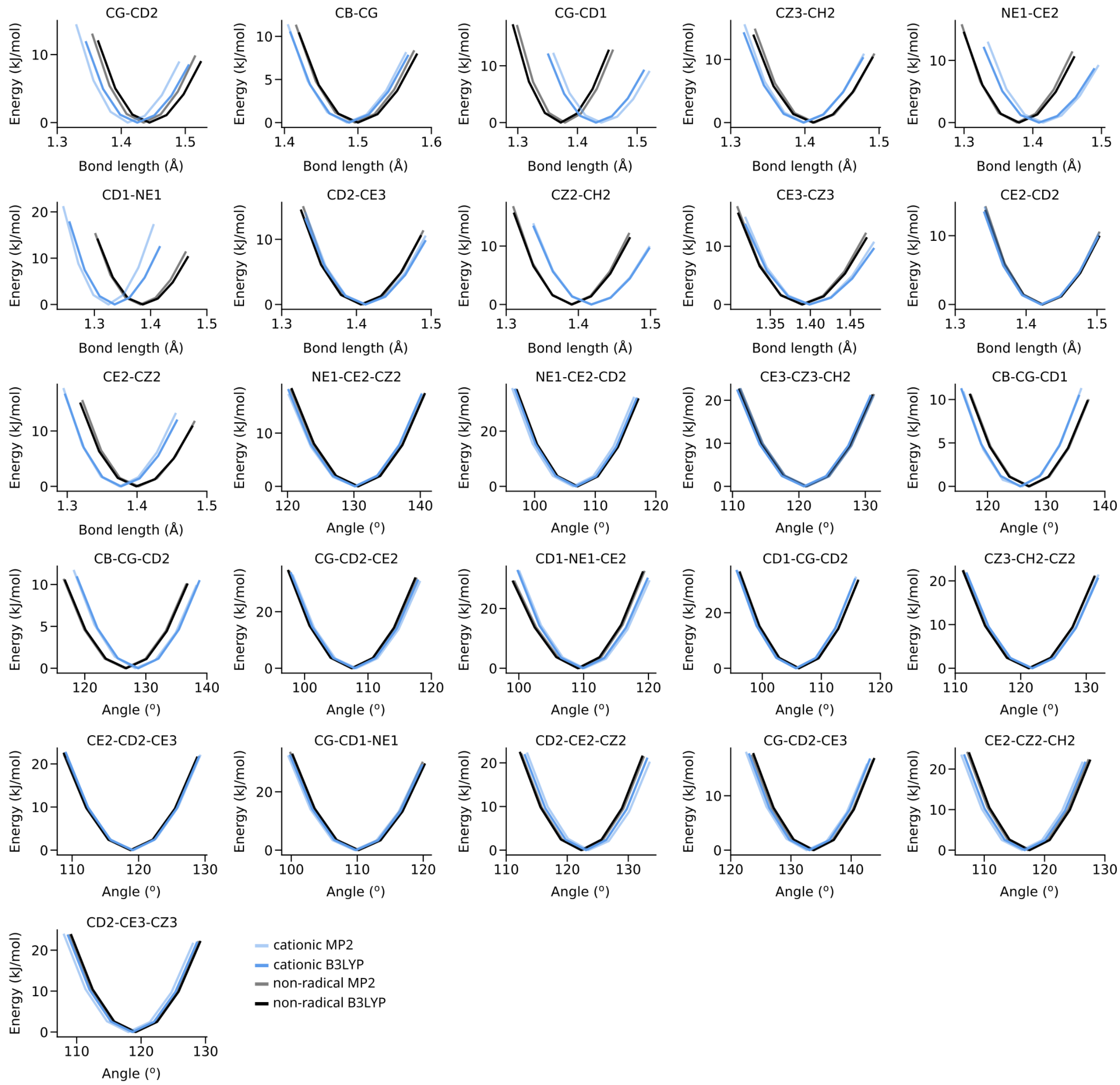

Figure S6: **Comparison of MP2 and DFT (B3LYP) PES for bonds and angles in 3-methylindole.** Shown are PES conducted for the non-radical (gray) and cationic radical (blue) states.

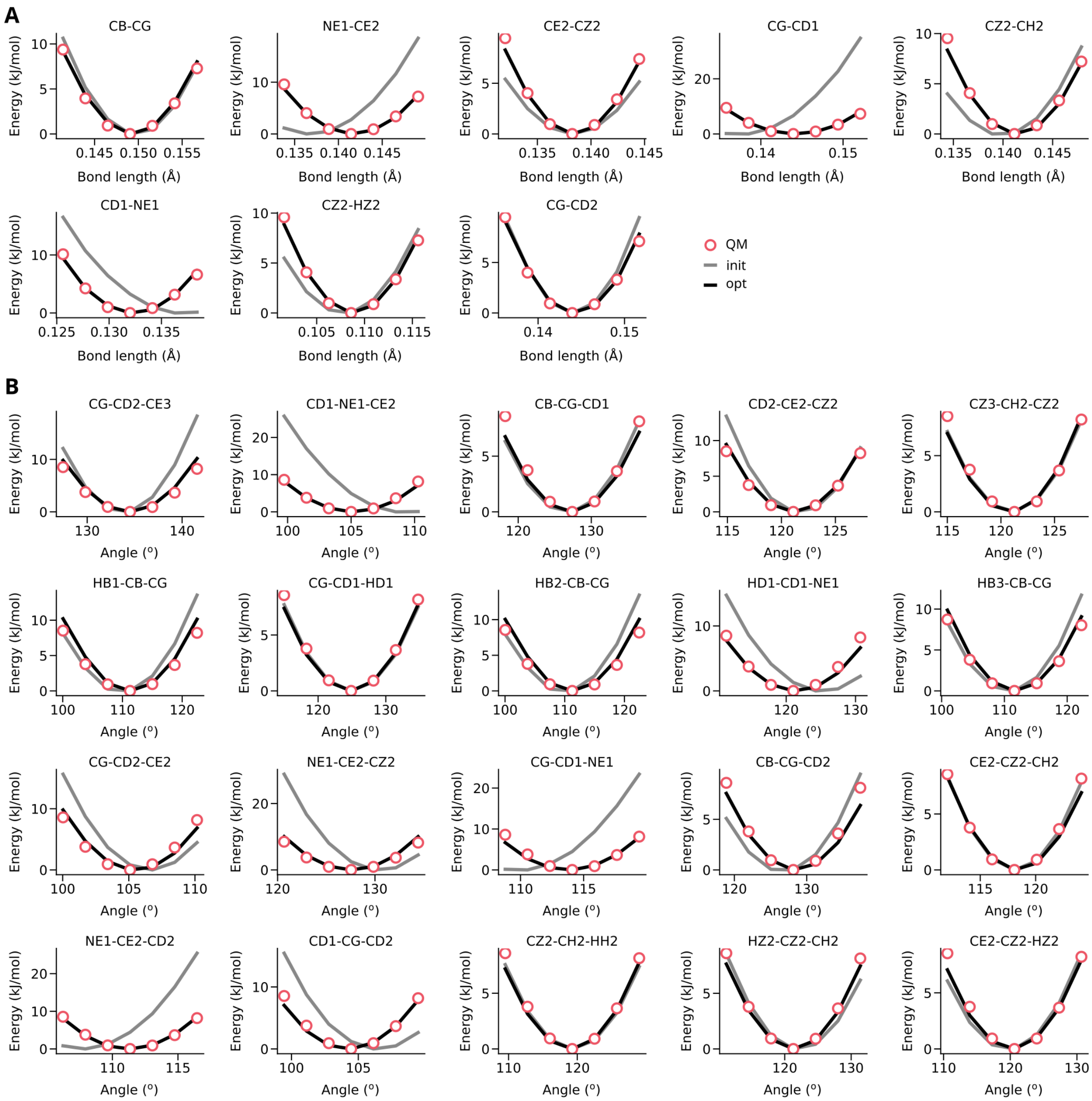

Figure S7: **Improved reproduction of QM PES with the optimized bond and angle parameters for TRP in the neutral radical state.** Shown are PES for bonds (**A**) and angles (**B**) conducted at the QM level of theory (red), and at the MM level of theory with the initial (gray) and optimized (black) sets of parameters.

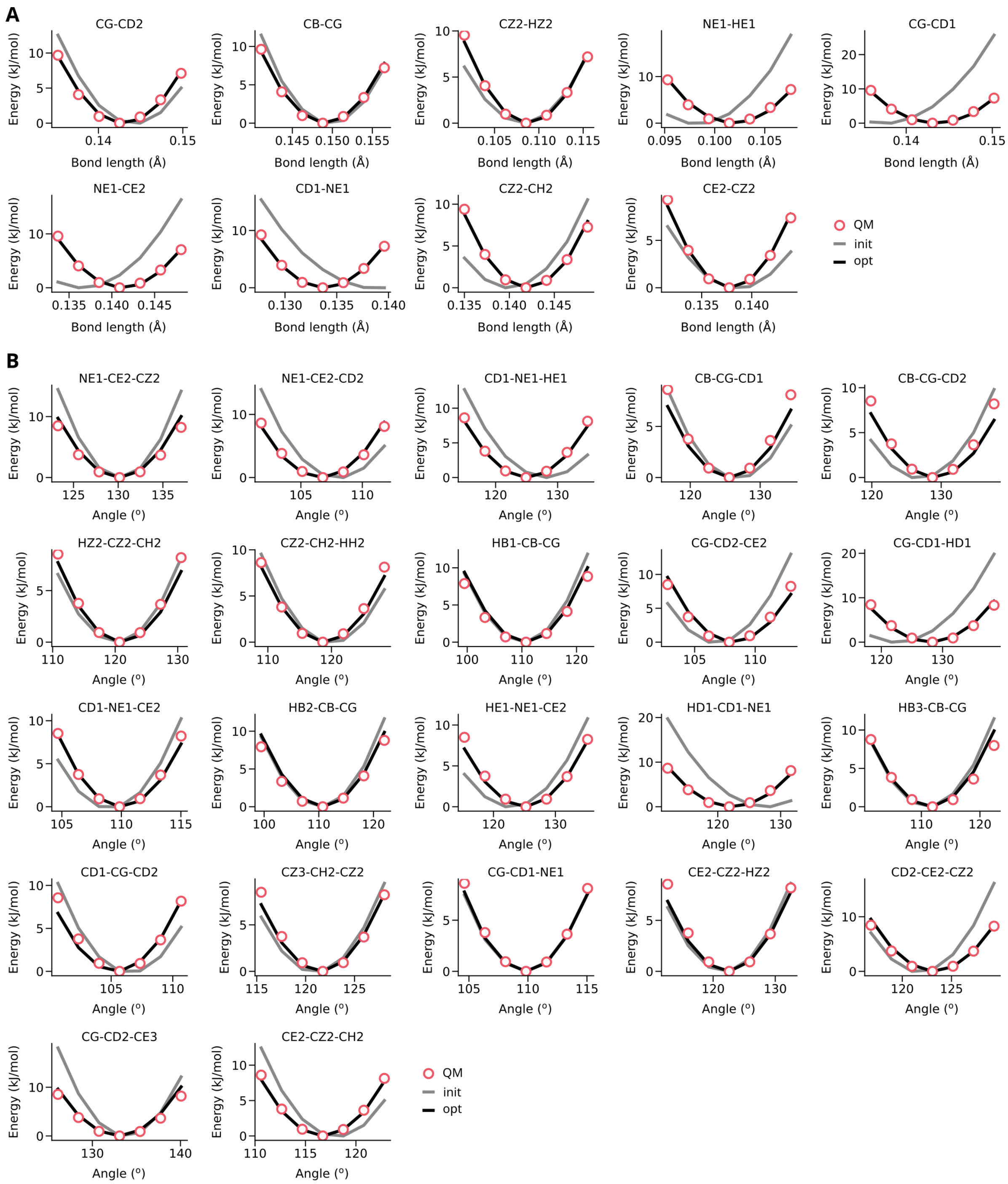

Figure S8: **Improved reproduction of QM PES with the optimized bond and angle parameters for TRP in the cationic radical state.** Shown are PES for bonds (**A**) and angles (**B**) conducted at the QM level of the theory (red), and at the MM level of theory with the initial (gray) and optimized (black) sets of parameters.

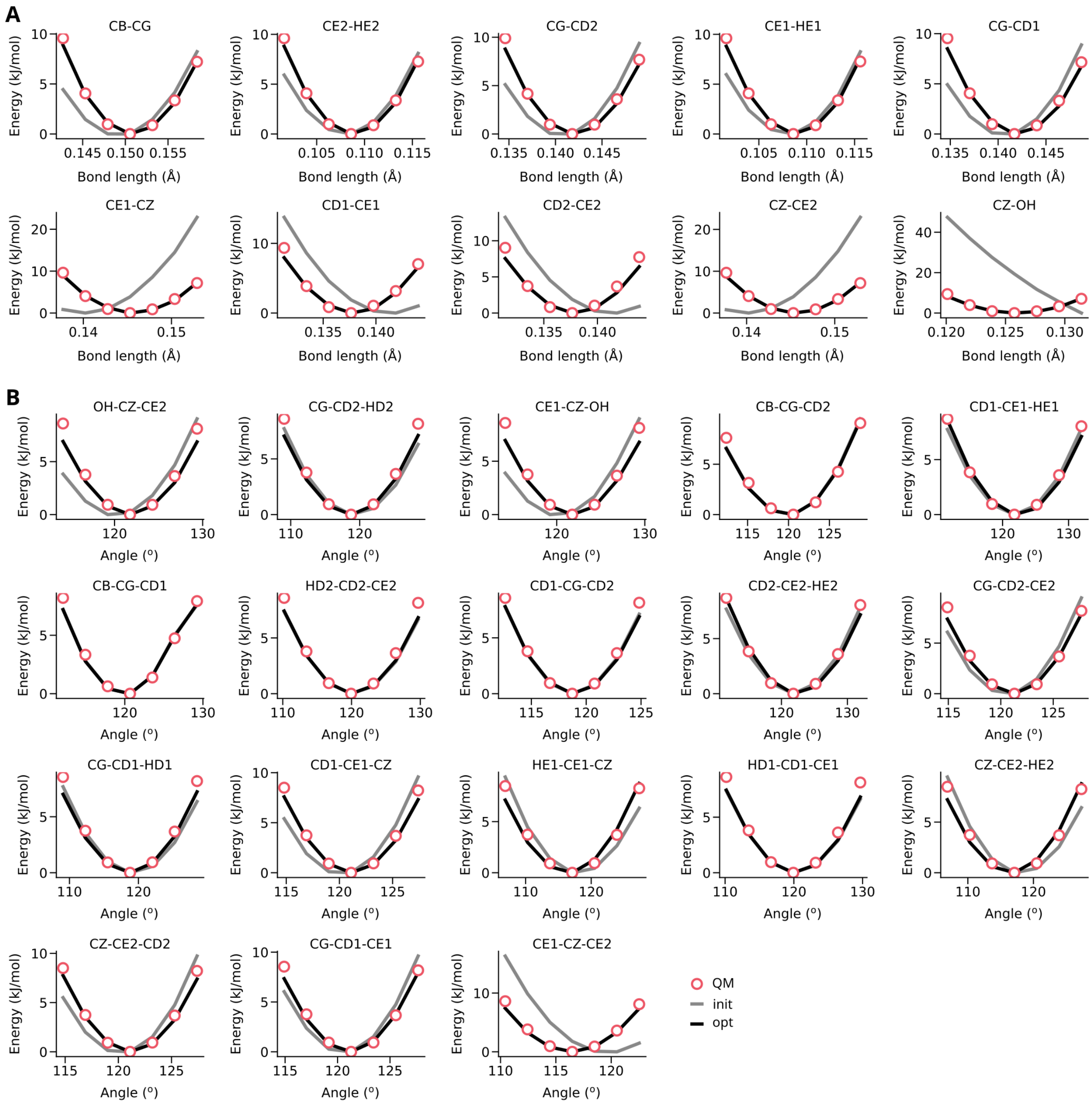

Figure S9: **Improved reproduction of QM PES with the optimized bond and angle parameters for TYR in the neutral radical state.** Shown are PES for bonds (**A**) and angles (**B**) conducted at the QM level of theory (red), and at the MM level of theory with the initial (gray) and optimized (black) sets of parameters.

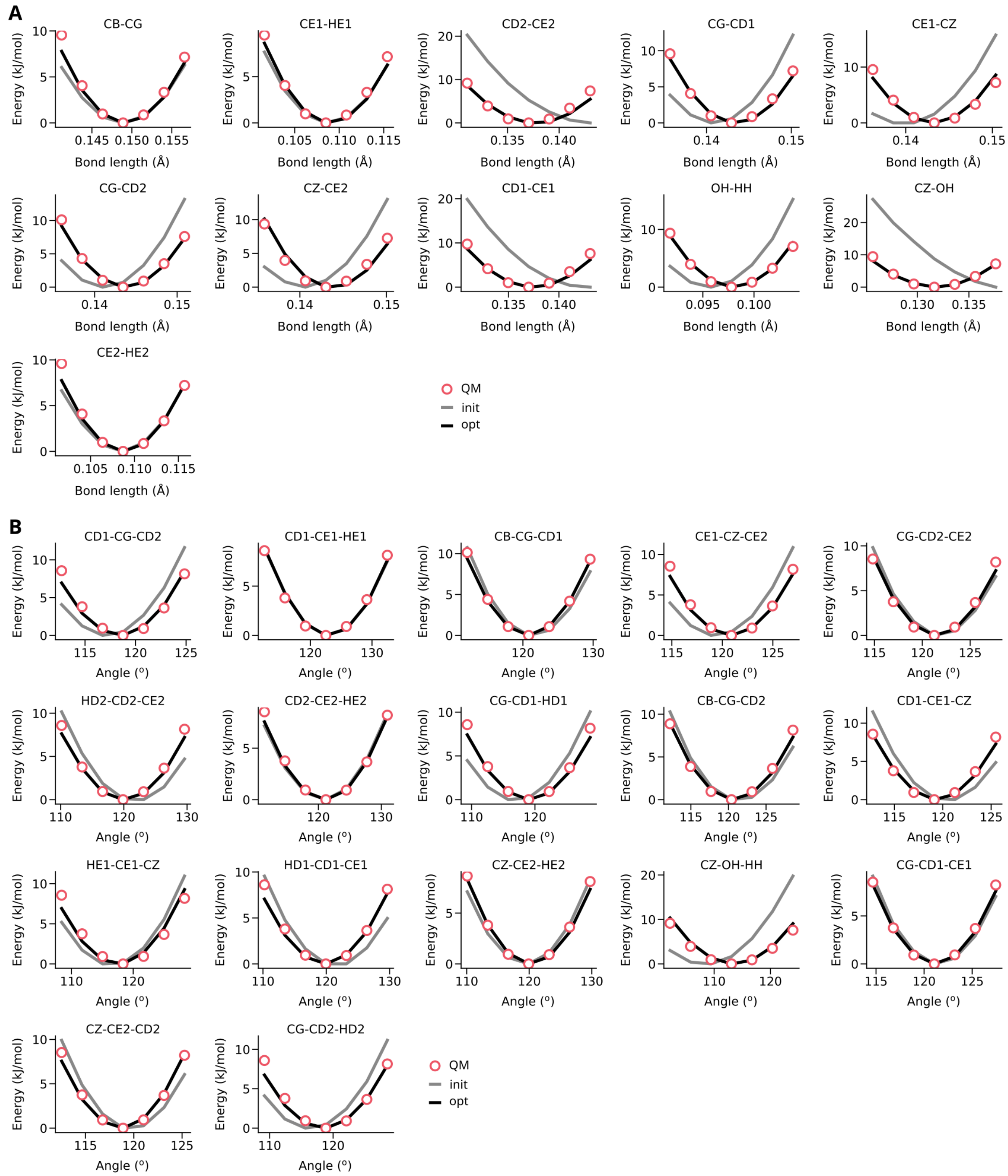

Figure S10: **Improved reproduction of QM PES with the optimized bond and angle parameters for TYR in the cationic radical state.** Shown are PES for bonds (**A**) and angles (**B**) conducted at the QM level of theory (red), and at the MM level of theory with the initial (gray) and optimized (black) sets of parameters.

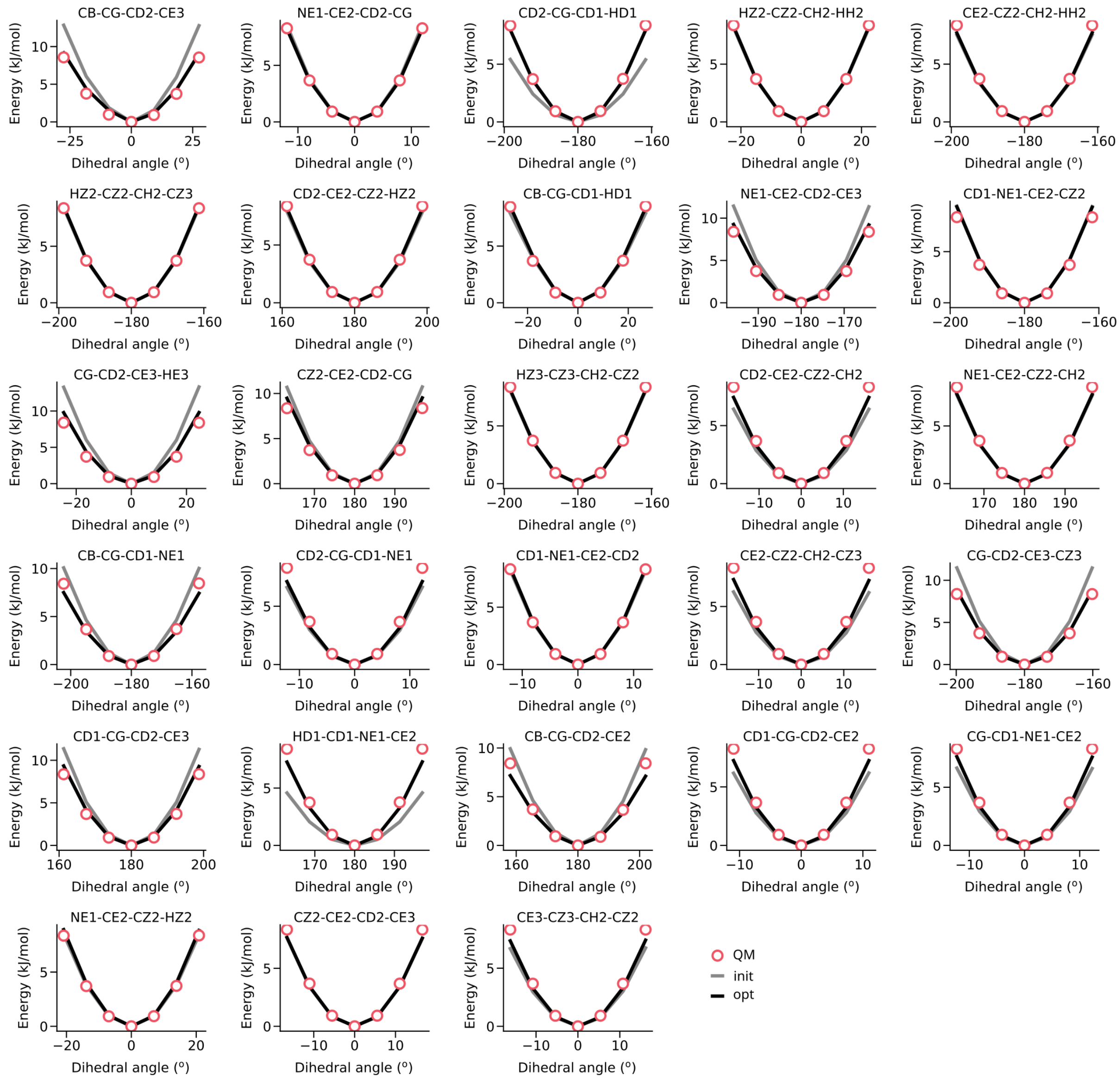

Figure S11: **Improved reproduction of QM PES with the optimized stiff-dihedral parameters for TRP in the neutral radical state.** Shown are PES conducted at the QM level of theory (red), and at the MM level of theory with the initial (gray) and optimized (black) sets of parameters.

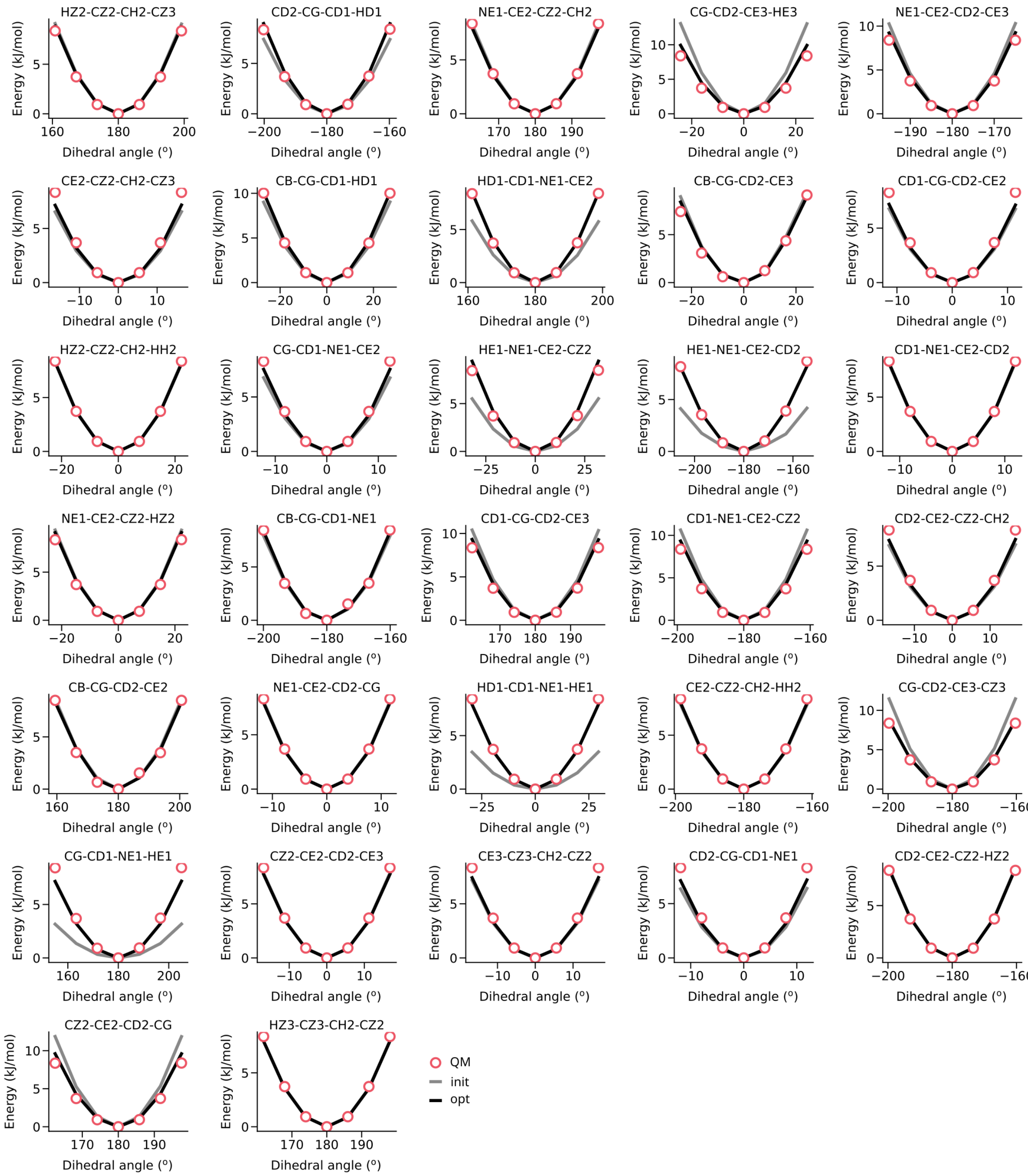

Figure S12: **Improved reproduction of QM PES with the optimized stiff-dihedral parameters for TRP in the cationic radical state.** Shown are PES conducted at the QM level of theory (red), and at the MM level of theory with the initial (gray) and optimized (black) sets of parameters.

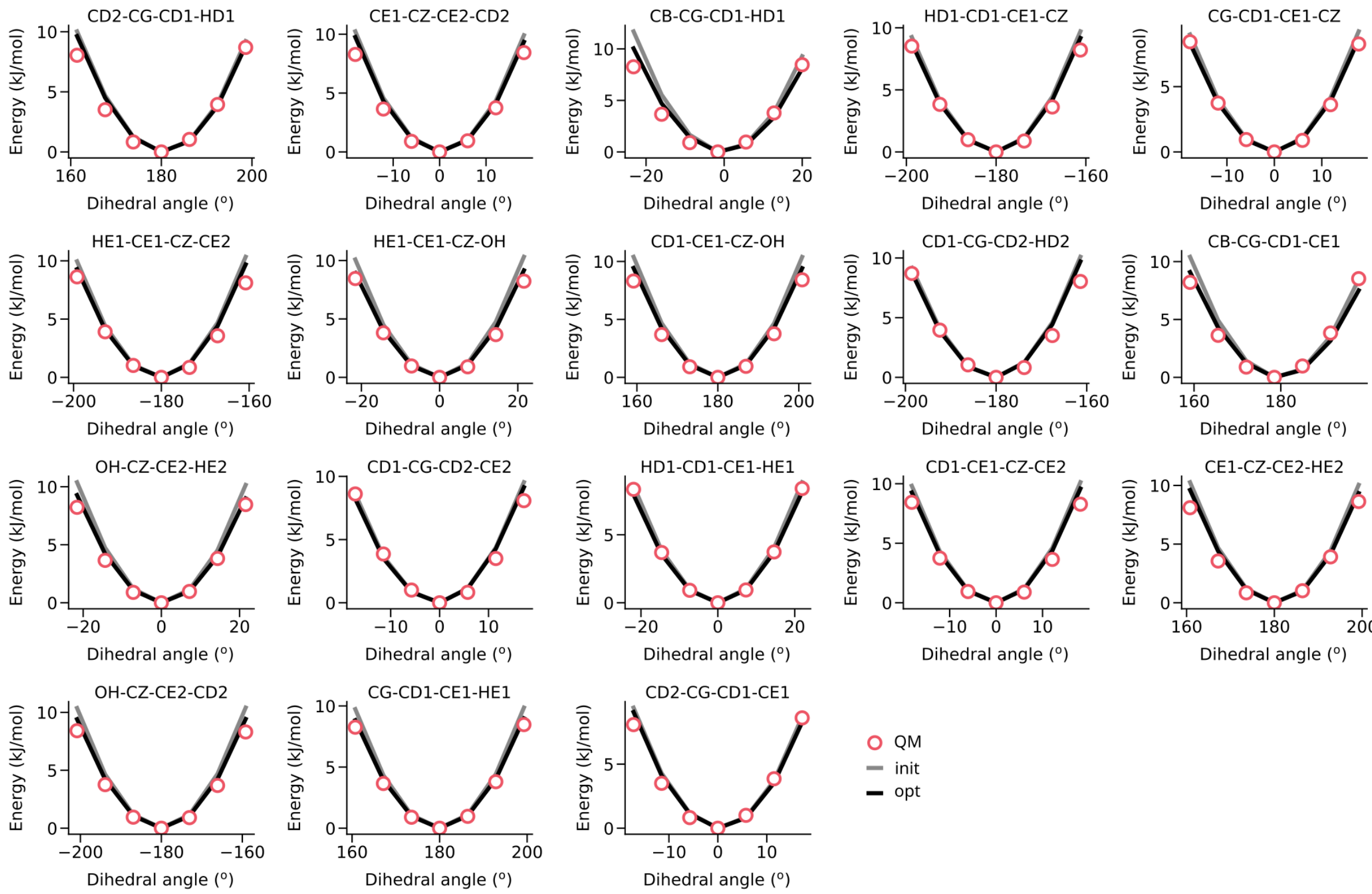

Figure S13: **Improved reproduction of QM PES with the optimized stiff-dihedral parameters for TYR in the neutral radical state.** Shown are PES conducted at the QM level of theory (red), and at the MM level of theory with the initial (gray) and optimized (black) sets of parameters.

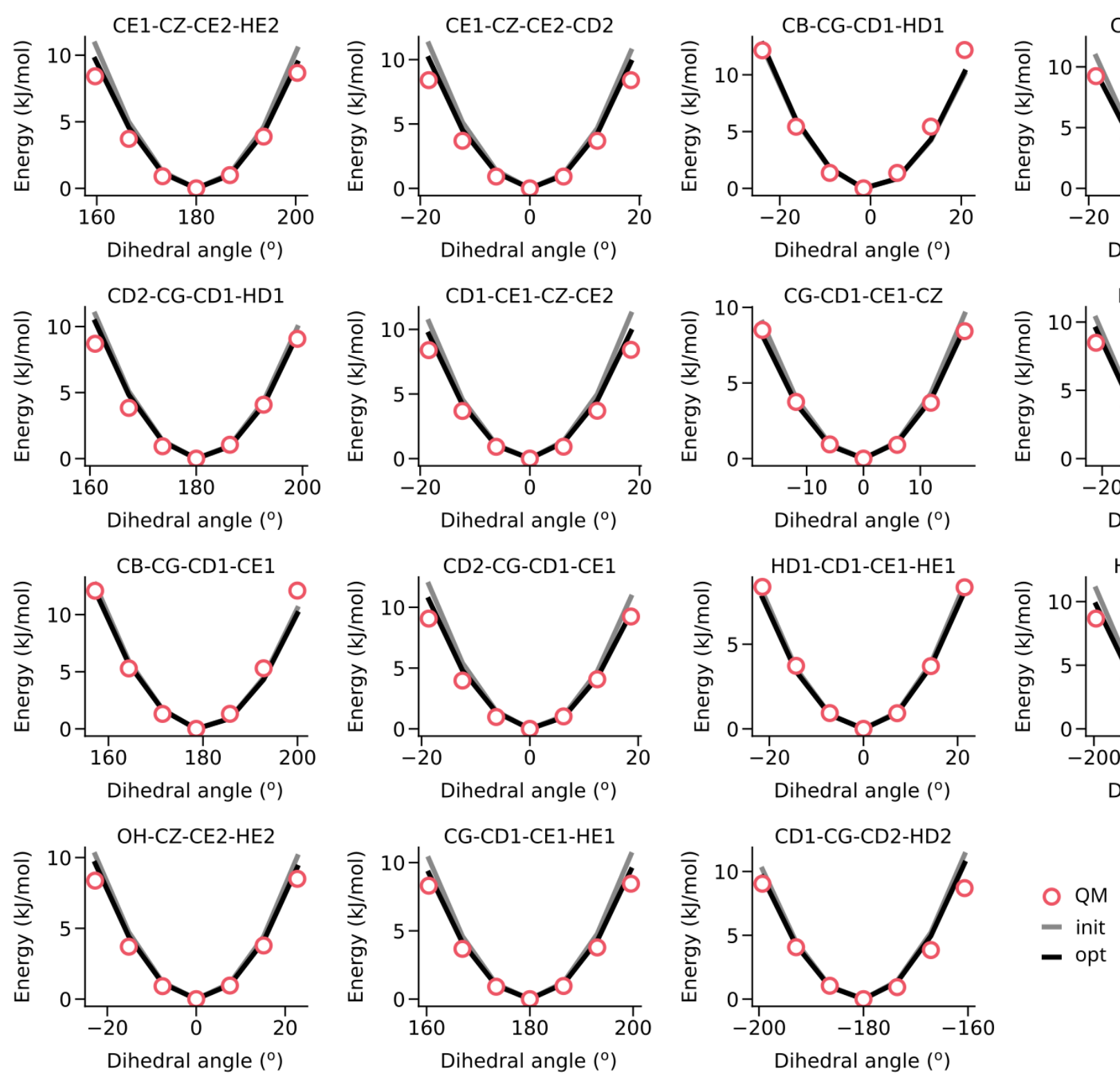

Figure S14: **Improved reproduction of QM PES with the optimized stiff-dihedral parameters for TYR in the cationic radical state.** Shown are PES conducted at the QM level of theory (red), and at the MM level of theory with the initial (gray) and optimized (black) sets of parameters.

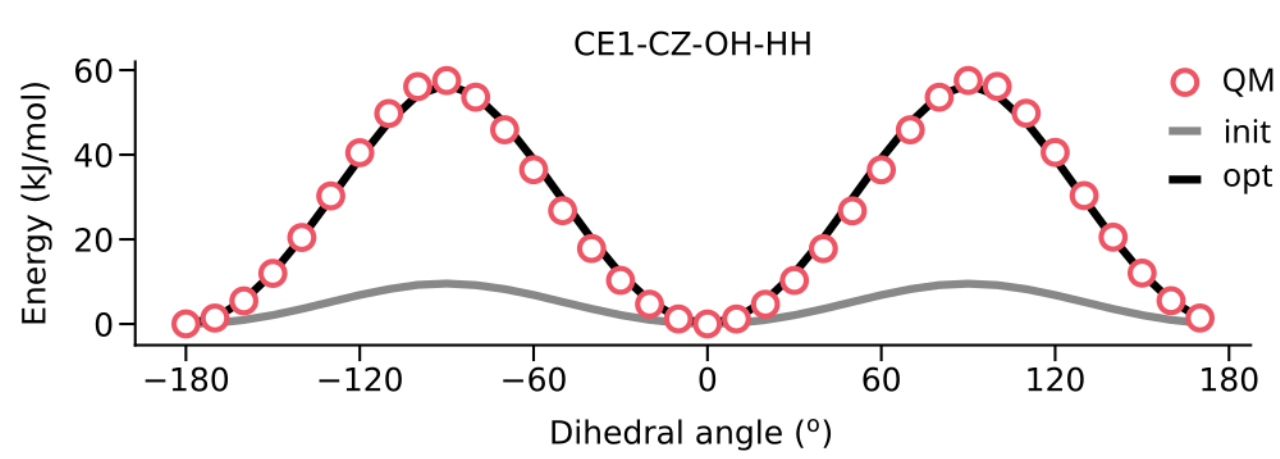

Figure S15: **Improved description of the hydroxyl rotation in the cationic radical state of the TYR residue.** Shown are PES conducted at the QM level of theory (red), and at the MM level of theory with the initial (gray) and optimized (black) sets of parameters.

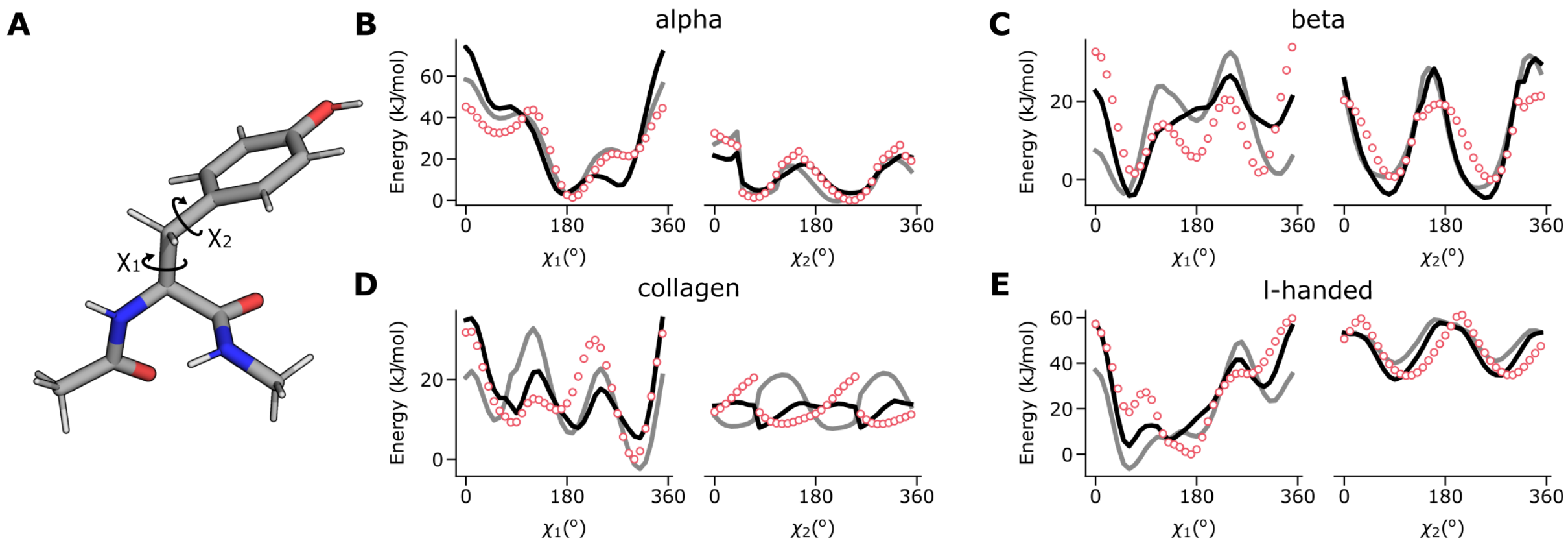

Figure S16: **Optimization of soft-dihedral parameters for the TYR dipeptide in the cationic radical state.** **A** TYR dipeptide in the cationic radical state with the indicated angles χ_1 and χ_2. **A** TRP dipeptide in the cationic radical state with the indicated angles χ_1 and χ_2. **B**, **C**, **D**, **E** MM potential energy scans along the χ_1 and χ_2 dihedral angles with the dipeptide backbone restrained to the *alpha* (**B**), *beta* (**C**), *collagen* (**D**), or *l-handed* (**E**) conformation. Shown are the scans conducted with the unadjusted (gray) and optimized (black) MM parameters. QM data are shown as red circles.

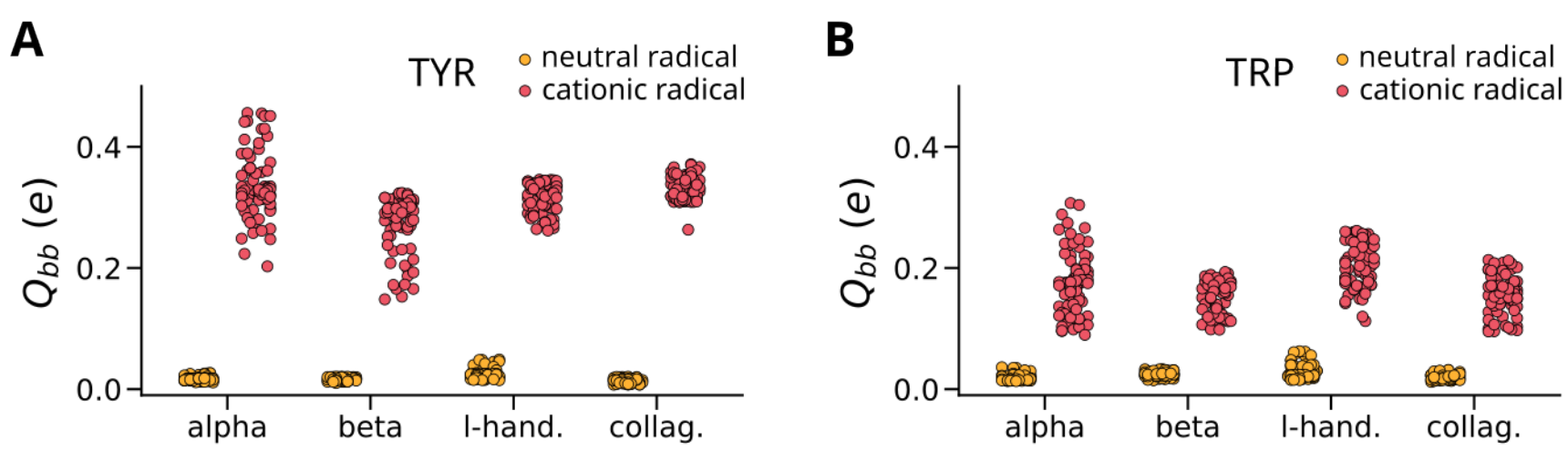

Figure S17: **A**, **B** Total charge (Q_{bb}) of the backbone and terminal methyl atoms in the dipeptide models of TYR (**A**) and TRP (**B**) in the neutral radical (orange) and cationic radical (red) states. The total charge was calculated as the sum of the atomic charges estimated using the Minimal Basis Iterative Stockholder method. Each data point corresponds to a single-point calculation on a dipeptide conformation extracted from the dihedral-angle scans with the indicated backbone conformation.

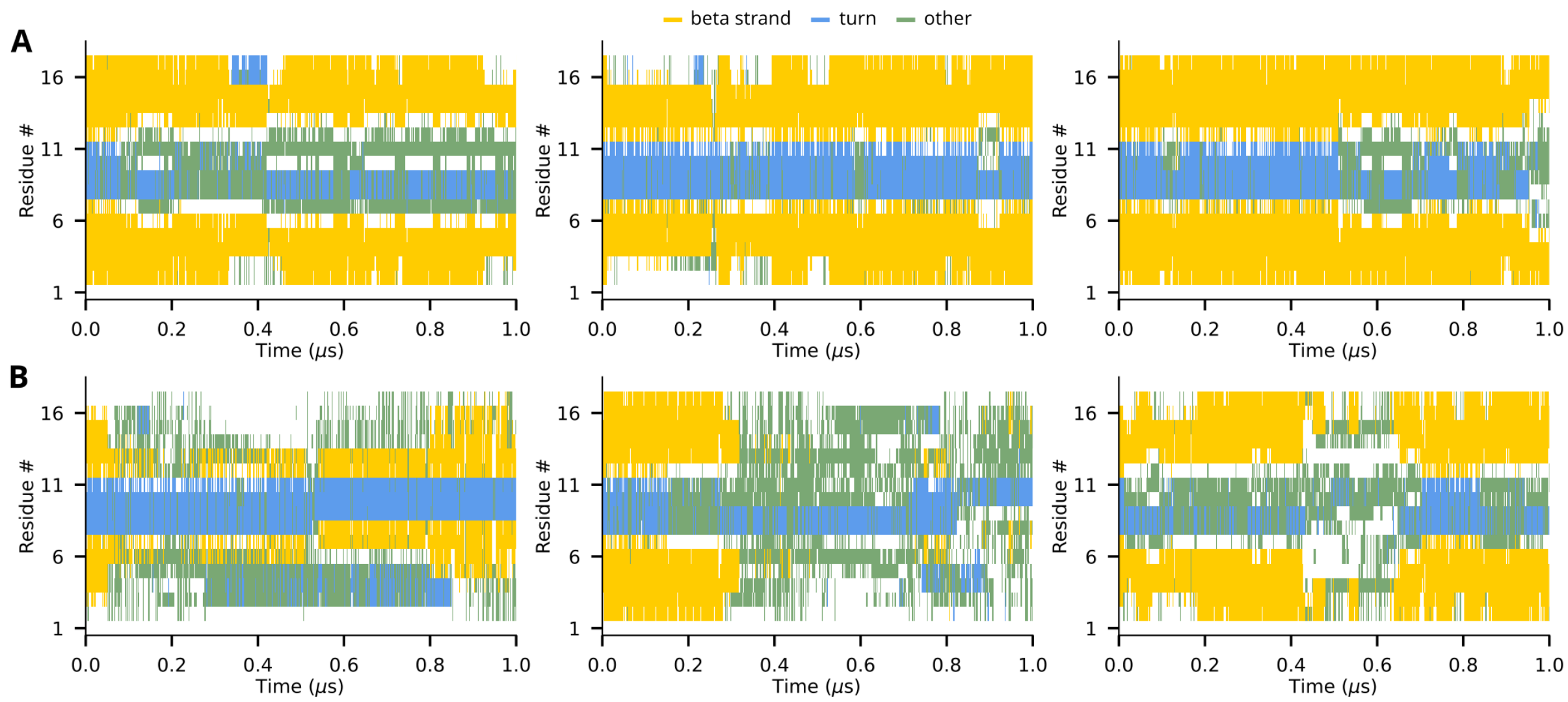

Figure S18: **Temporal evolution of the secondary structure of peptide A. A**, **B** Temporal evolution of the secondary-structure assignment in MD simulations with Y5 in the non-radical (**A**) and neutral radical (**B**) states. β strands, turns, and other secondary structures are shown in gold, blue, and green, respectively, and unstructured regions are shown in white.

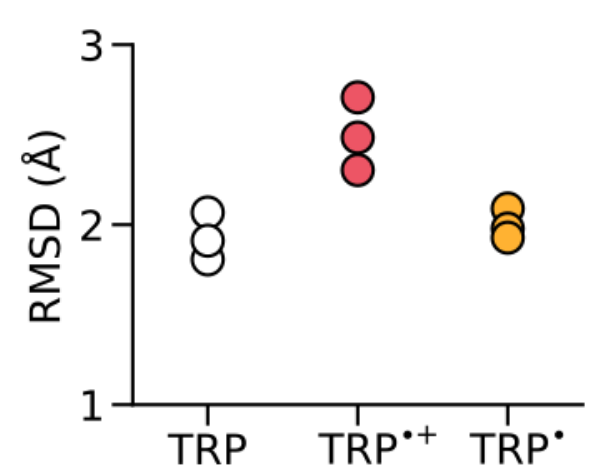

Figure S19: **Stability of α3W in simulations with W34 in different redox states.** The root-mean square deviation (RMSD) of the Cα atoms in MD simulations of α3W with W34 in the non-radical, cationic radical, and neutral radical states.

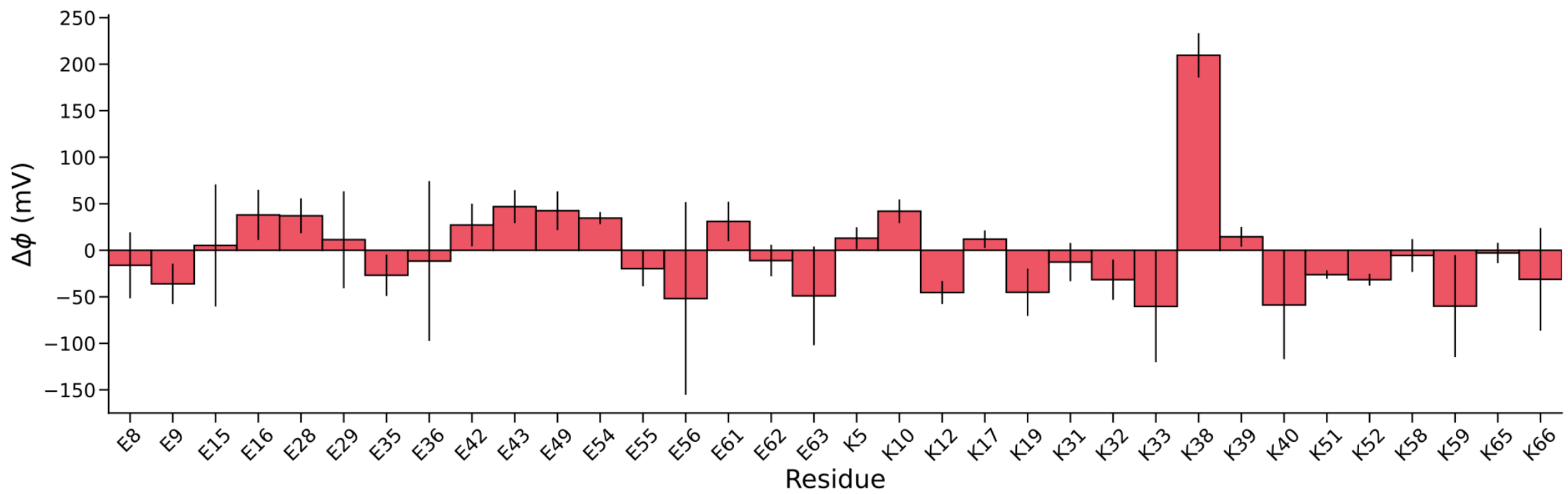

Figure S20: **Change in the electrostatic potential of α3W with W34 in the cationic radical state.** The electrostatic potential was measured on the carboxyl and amino groups of the GLU and LYS residues, respectively. Shown is the difference in the electrostatic potential sensed by the selected residues with W34 in the cationic radical compared to the non-radical state. Error bars indicated the standard deviation across three independent simulation replicas.

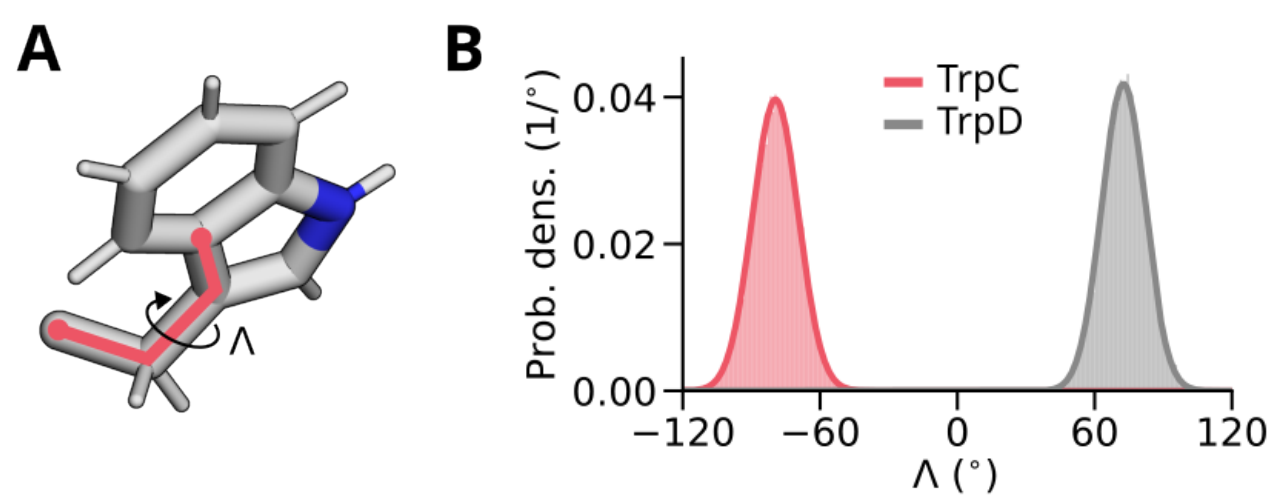

Figure S21: **Fluctuations in the TRP dihedral angle Λ in ClCry4. A** The dihedral angle Λ used for the analysis. **B** Distribution of the dihedral angle (Λ) in simulations of ClCry4 with TrpC (red) or TrpD (gray) in the cationic radical state. Fitting of the underlying histograms with the Gaussian function is shown by solid lines.

Table S1: **Librational motions of the TrpC and TrpD residues of ClCry4 in the cationic radical state.** Shown are the scaling parameters of the Rayleigh distributions used to fit the data on the fluctuations of the principle axes of the indole group.

axis	TrpC	TrpD
x	6.3°	6.8°
y	5.9°	6.8°
z	6.6°	7.1°
